# Strongly Modified Mechanical Properties and Phase Transition in AlPO_4_-17 Due to Insertion of Guest Species at High Pressure

**DOI:** 10.1021/acs.jpcc.3c03513

**Published:** 2023-07-18

**Authors:** Frederico G. Alabarse, Benoît Baptiste, Yoann Guarnelli, Boby Joseph, Julien Haines

**Affiliations:** † Elettra Sincrotrone Trieste, Trieste 34149, Italy; ‡ Institut de Minéralogie, de Physique des Matériaux et de Cosmochimie, (IMPMC), UMR 7590 CNRS—Sorbonne Université—IRD—MNHN, 4 place Jussieu, 75252 Paris, Cedex 5, France; § Institut Charles Gerhardt Montpellier, CNRS, Université de Montpellier, ENSCM, 34293 Montpellier, France

## Abstract

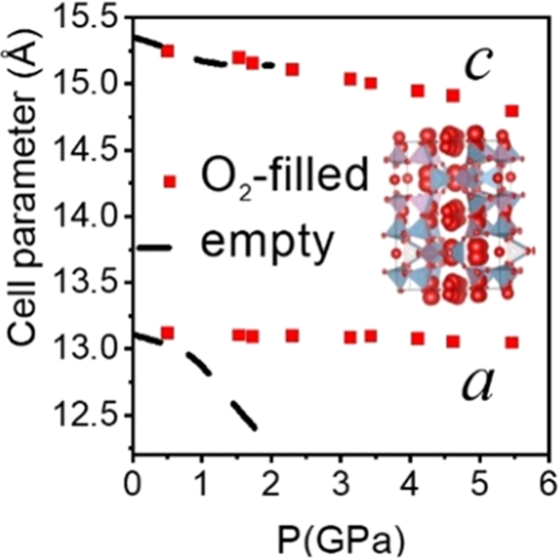

The porous aluminophosphate AlPO_4_-17 with a hexagonal erionite structure, exhibiting very strong negative thermal expansion, anomalous compressibility, and pressure-induced amorphization, was studied at high pressure by single-crystal and powder X-ray diffraction in the penetrating pressure transmitting media N_2_, O_2_, and Ar. Under pressure, these guest species were confirmed to enter the pores of AlPO_4_-17, thus completely modifying its behavior. Pressure-induced collapse in the *xy* plane of AlPO_4_-17 no longer occurred, and this plane exhibited close to zero area compressibility. Pressure-induced amorphization was also suppressed as the elastic instability in the *xy* plane was removed. Crystal structure refinements at a pressure of 5.5 GPa indicate that up to 28 guest molecules are inserted per unit cell and that this insertion is responsible for the reduced compressibility observed at high pressure. A phase transition to a new hexagonal structure with cell doubling along the **a** direction was observed above 4.4 GPa in fluid O_2_.

## 1 Introduction

**Figure 1:**
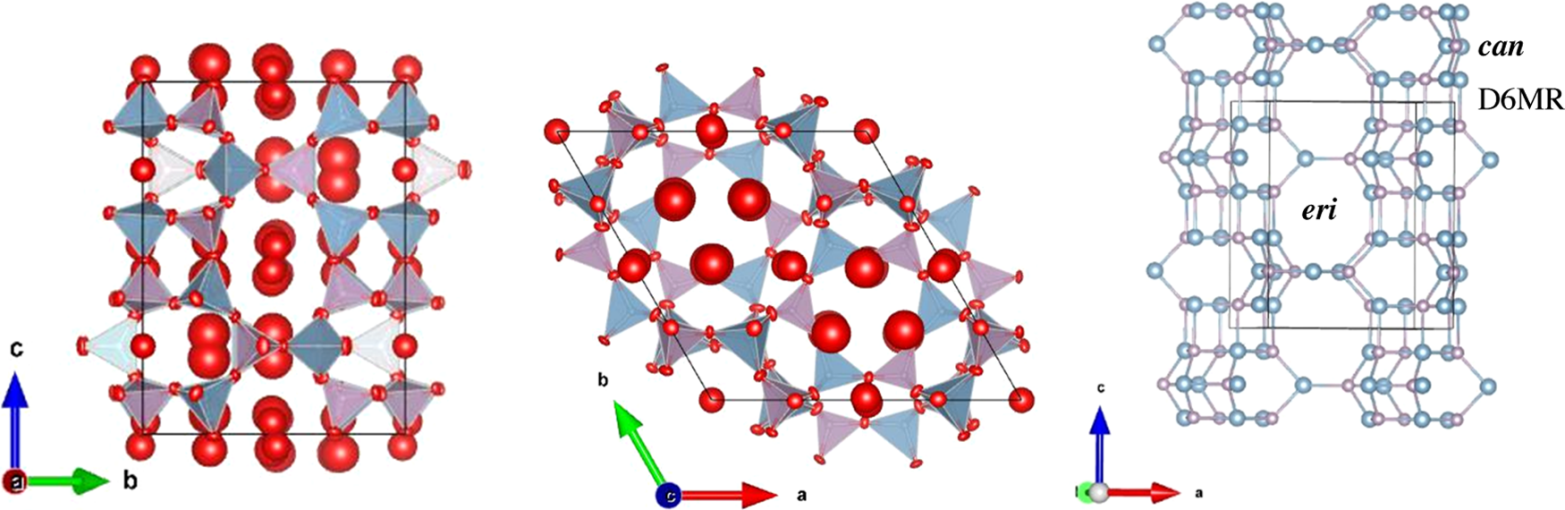
Projections (left, center) of the crystal structure of O_2_–filled AlPO_4_-17 at 1.5 GPa. The tetrahedra correspond to the AlO_4_ and PO_4_ units of AlPO_4_. Atomic displacement ellipsoids for O_2_ (red) are plotted at 50% probability. Note the larger atomic displacement for the oxygen atoms in the inserted O_2_ molecules as compared to those in the tetrahedra. Ring structures (*eri*, *can*, D6MR) are displayed (on the right) without the oxygen atoms.

The porous aluminophosphate AlPO_4_-17, which is isostructural with the zeolite erionite (space group P63/*m*), exhibits the highest negative thermal expansion^1,2^ in the zeolite class of materials. The erionite structure of AlPO_4_-17 (Figure 1) is characterized by 4-, 6-, 8-, and 12-membered rings (MR) of tetrahedra forming large erionite cages (*eri*), with a diameter of 6.556 Å, and columns of smaller cancrinite cages (*can*) linked to double six-membered rings (D6MR) along *z*. The negative thermal expansion in this material is linked to the thermally excited transverse motion of oxygen atoms bridging the AlO_4_ and PO_4_ tetrahedra, which bring the Al and P atoms closer together.^2^

Atoms or molecules with a maximum kinetic diameter of about 3.4 Å can diffuse into the porosity of the AlPO_4_-17 structure via the 8-membered rings. The negative thermal expansion in AlPO_4_-17 was found to be tuned by the insertion of guest molecules at high pressure giving close to zero thermal expansion in the *xy* plane.^3^

In addition to negative thermal expansion, AlPO_4_-17 also exhibits uncommon mechanical properties at high pressure. This material exhibits an elastic instability, becoming increasingly softer in the *xy* plane upon compression.^4^ Similar behavior has also been observed in zeolites,^5^ cyanides,^6,7^ and metal–organic frameworks.^8^ In the case of AlPO_4_-17,^4^ this instability gives rise to complete amorphization of the material at modest pressures of just above 2 GPa, which is lower than that observed for many zeolites^9−20^ and aluminophosphates.^21−23^ These mechanical properties could also be expected to be modified by pore filling. Thus, in the present study, nitrogen, oxygen, and argon with kinetic diameters^24^ of 3.64, 3.46, and 3.40 Å, respectively, which are close to or slightly larger than the maximum size of 3.4 Å, were selected to be inserted in AlPO_4_-17 under high pressure in order to study guest insertion and to determine its effect on the compressibility and phase stability of this material.

## 2 Experimental Methods

Single crystals of hydrated AlPO_4_-17 with maximum dimensions of 250 × 70 × 70 μm^3^ were synthesized as described previously^4,25^ from aluminum triisopropoxide and phosphoric acid using *N*,*N*,*N*′,*N*′-tetramethyl-1,6-hexanediamine as a structure directing agent. The crystals were calcined in air at 500 °C for 24 h.

AlPO_4_-17 single crystals (maximum dimensions 190 × 70 × 70 μm^3^) or ground single crystals were placed in 235–300 μm diameter and 80–110 μm thick copper or stainless steel gaskets along with a ruby pressure calibrant in membrane diamond anvil cells with opening angles of between 50 and 100°. The DACs were placed in a cryogenic gas loading system, and the sample was dehydrated for 2 h at 110 °C under vacuum (4 Pa). Nitrogen, oxygen, or argon was then loaded cryogenically by condensing the corresponding gases.

X-ray diffraction measurements (λ = 0.4957 Å) under pressure were performed with an 80 μm beam on the Xpress beamline equipped with a PILATUS3 S 6 M (DECTRIS) detector at the Elettra Sincrotrone Trieste (Trieste, Italy). The detector was placed between 249 and 280 mm from the sample for the single-crystal measurements at 621–951 mm for the powder runs. The pressure was measured based on the shift in the R_1_ fluorescence line of ruby.^26^

For the powder studies, the XRD images were converted to 1-D diffraction profiles using Dioptas.^27^ Le Bail (Figures S1 and S2) and Rietveld refinements were performed using FullProf.^28^ Le Bail and Rietveld refinements gave identical cell parameters; however, there are too many free atomic fractional coordinates to get accurate interatomic distances and angles for AlPO_4_-17.

Diffraction data were collected from the AlPO_4_-17 single crystals using phi scans from −30 to +30° or −45 to +45°, depending on the opening angle of the DAC. Data reduction was performed with CrysAlisPro 1.171.39.46 (Rigaku OD, 2018). The crystal structure was refined using Shelxl-2017/1^29^ with the WinGX^30^ and OLEX^31^ interfaces. The Squeeze method^32^ was applied using the OLEX interface. Crystal structures were plotted using Vesta.^33^

## 3 Results and Discussion

**Figure 2:**
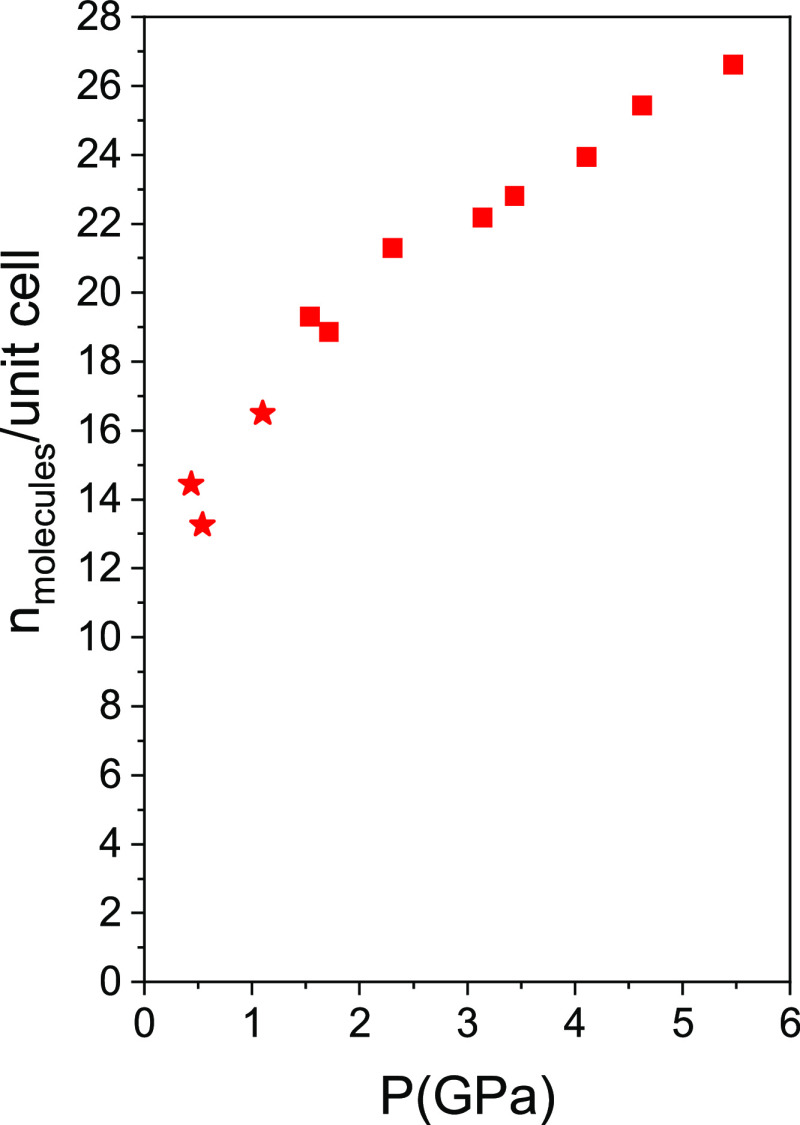
Guest content for oxygen-filled AlPO_4_-17 as a function of pressure as obtained using the Squeeze method. The points at 0.43, 0.54, and 1.1 GPa (stars) were obtained on decompression.

Synchrotron X-ray diffraction studies were performed on both single crystals and powders of AlPO_4_-17 in N_2_, O_2_, and Ar. In the pressure range studied up to 6 GPa, good single-crystal XRD data were limited to AlPO_4_-17 in O_2_ due to its higher solidification point of 5.5 GPa. Solidification of N_2_ and Ar occurs at much lower pressures of 2.4 and 1.5 GPa, respectively. Insertion of O_2_ in AlPO_4_-17 was clear from Fourier difference maps (Figure S3). Refining the structures with guest atoms on fully occupied sites based on the peaks on the Fourier difference maps yielded R1 agreement factors in the range of 5.4–11.5%. This type of model, Figure 1, resulted in large atomic displacement parameters for the guest atoms as in ref (3), implying significant disorder, probably both positional and orientational, and corresponds to filling of the pores with 24 guest molecules per unit cell. In order to better quantify the degree of filling by the guest, the Squeeze method^32^ was applied to determine the number of electrons and thus the number of guest molecules in the pores. This was found to be reliable only for a relatively large single-crystal 125 μm × 50 μm × 50 μm^3^ in oxygen, for which the completeness was typically between 93 and 95% rather than 75% for other crystals investigated. This method resulted in a significant decrease in the *R*1 values to 2.8–4.3%. The obtained pore content per unit cell (Figure 2) was found to increase up to 28 molecules in the pressure range up to 4 GPa. This was slightly higher than the amount of H_2_O molecules found in the large erionite cages of hydrated AlPO_4_-17, for example (22 H_2_O/uc).^25,26^

**Figure 3:**
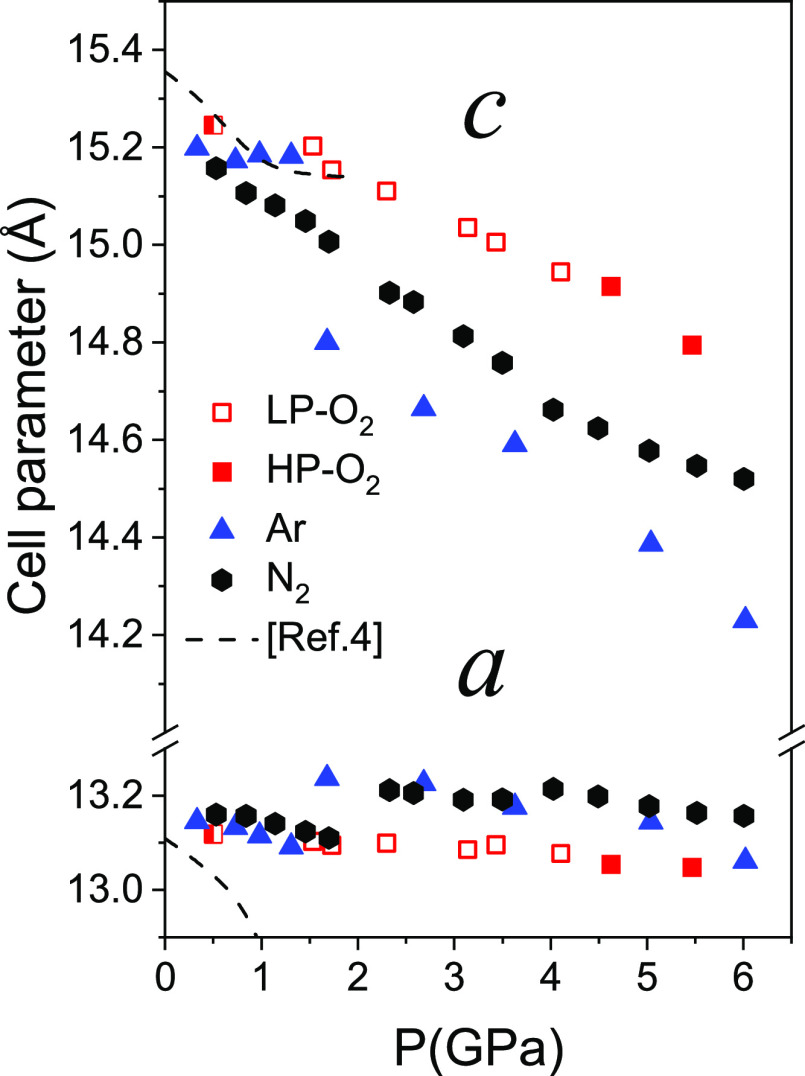
Unit cell parameters of guest-filled AlPO_4_-17 as a function of pressure. In the case of the high-pressure phase of O_2_-filled AlPO_4_-17 (filled squares), *a*/2 is plotted in order to directly compare the values to those of the low-pressure phase. Data for empty AlPO_4_-17 are from ref (4). Data at 0.5 GPa (half-filled square) for AlPO_4_-17 in O_2_ are from ref (3).

**Figure 4:**
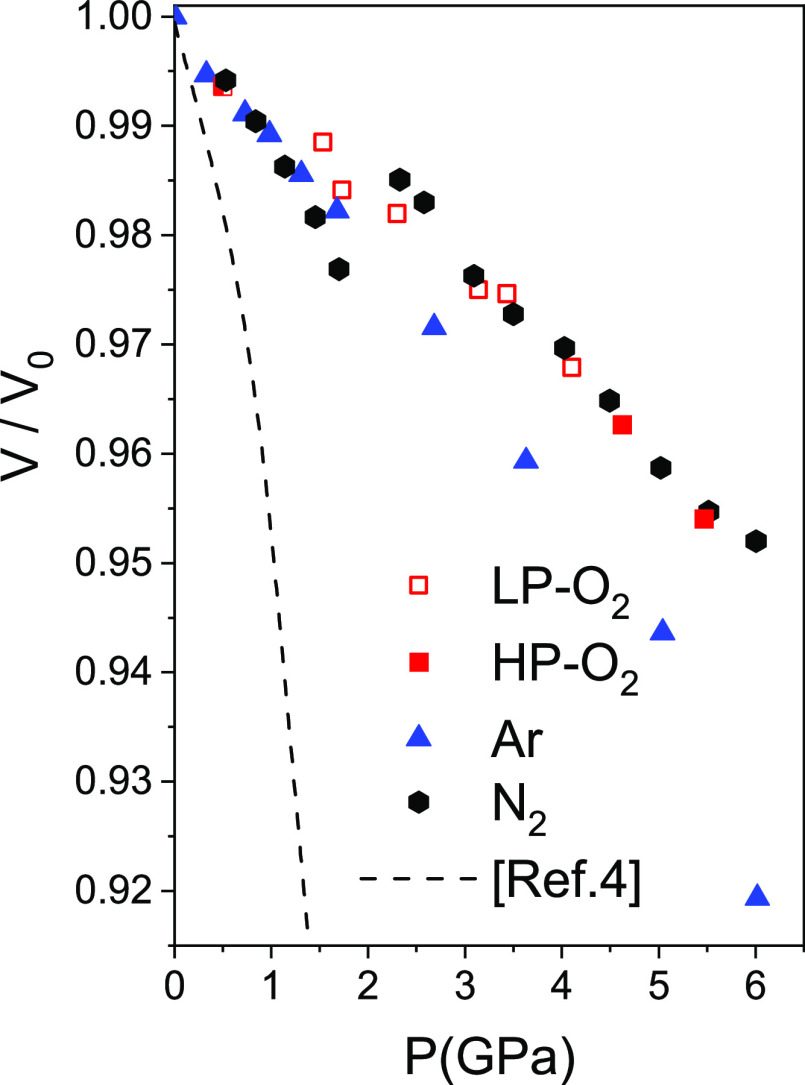
Relative volume of guest-filled AlPO_4_-17 as a function of pressure. Data for empty AlPO_4_-17 are from ref (4). The point at 0.5 GPa for AlPO_4_-17 in O_2_ is from ref (3).

This pore filling has major effects on the linear and volume compressibilities (Figures 3 and 4, Tables S1–S3) as compared to the intrinsic behavior of AlPO_4_-17 in the nonpenetrating pressure transmitting medium, silicone oil.^4^ AlPO_4_-17 intrinsically presents an elastic instability, which manifests itself by an increasing collapse in the *xy* plane around the empty pores giving rise to a negative pressure derivative of the bulk modulus. Compression along *z* follows a normal behavior. It can be seen that the major effect of filling with guest atoms and molecules is to suppress the anomalous behavior in the *xy* plane due to filling of the pores. The *a* cell parameter initially is stable or slightly increases depending on the guest and then begins to compress slightly (see Figure 3). The *c* parameter decreases initially both in empty AlPO_4_-17 and in the filled systems. The behavior in argon appears to be strongly affected by the solidification of the fluid at 1.5 GPa. A very strong discontinuous decrease in *c*, along with a marked increase in *a*, occurs at this pressure, which can be linked to the appearance of nonhydrostatic stress. The effect of nonhydrostratic stress was also observed previously on compressing monoclinic, hydrated AlPO_4_-17 in H_2_O^34^ with discontinuities in the pressure dependence of the cell parameters and volume at the solidification pressure of H_2_O at 0.9 GPa. In N_2_ in the fluid state, a discontinuous increase in *a* is observed, giving rise to increases in volume, Figure 4. In N_2_, above the pressures at which the volume increase occurs, and in O_2_, compression principally occurs along *c*, with *a* being very stiff. This is similar to what has been observed as a function of temperature when AlPO_4_-17 is filled with O_2_^3^, for which the thermal expansion along *a* is essentially zero, and strong negative thermal expansion is observed along *c*. The area compressibilities in the *xy* plane are 1.2(2) TPa^–1^ in O_2_ below the phase transition (see below) and 1.8(4) TPa^–1^ in N_2_ (above the volume jump) and are statistically zero over a range of at least 2 GPa. This essentially zero area compressibility is rare and is a phenomenon observed in relatively few materials, such as fluoroborates,^35^ iodates,^36^ and metal–organic frameworks.^37^ The structural flexibility is restricted to the **c** direction. Upon decompression (Tables S1 and S2), the *c* lattice parameter increases very strongly, and *a* actually decreases. This behavior could be related to differences in the O_2_ content.

**Figure 5:**
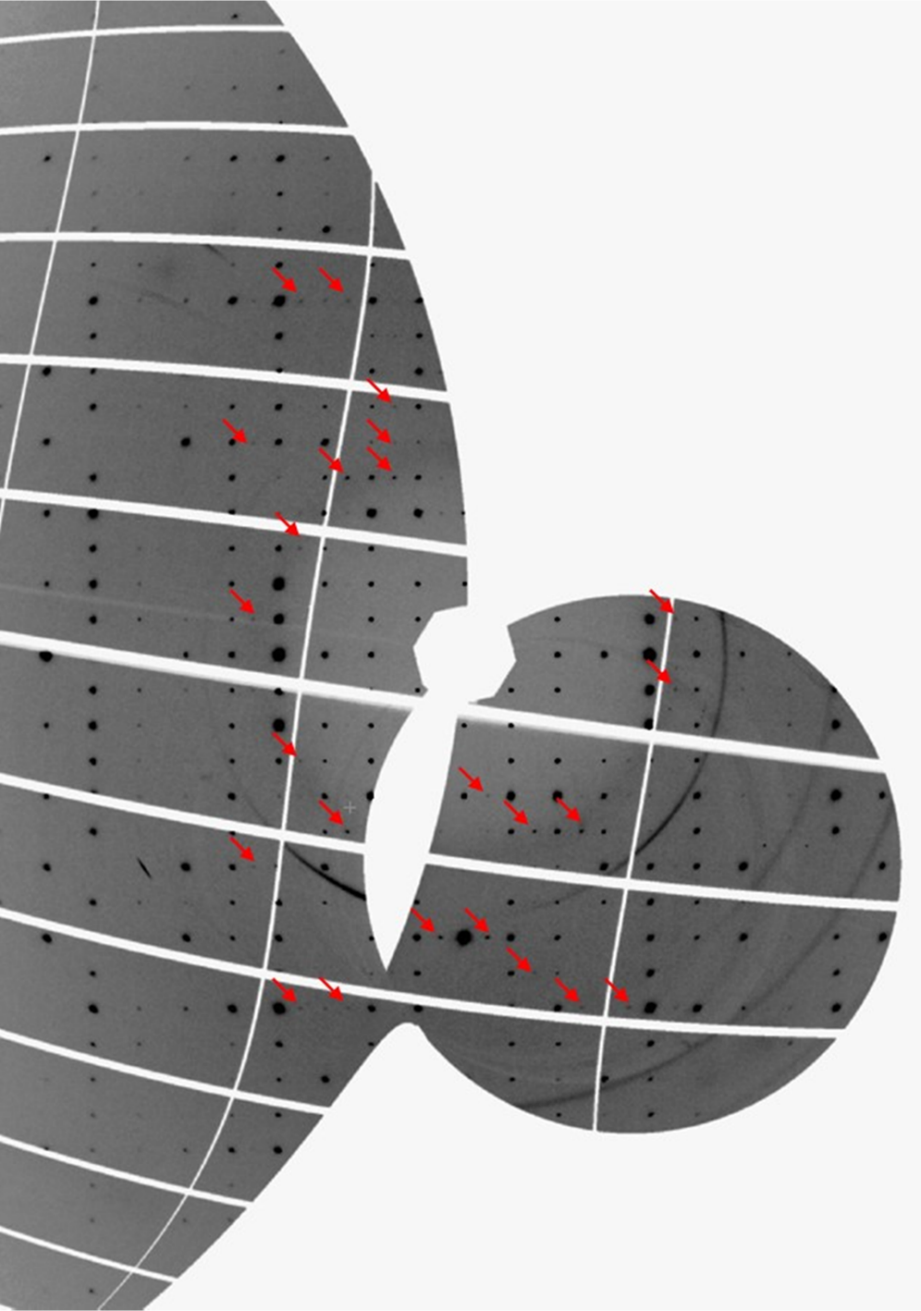
(0*kl*) Reciprocal space reconstruction for AlPO_4_-17/O_2_ at 5.5 GPa. Arrows indicate the principal superlattice reflections.

In the case of AlPO_4_-17 in O_2_, which remains fluid up to 5.5 GPa, a phase transition occurs just above 4.1 GPa. A series of superlattice reflections of the type 0 0.5 8, 0 0.5 5, and −0.5 1.5 5, for example, with a maximum relative intensity of 0.25%, appeared at the phase transition (Figure 5). This is consistent with a doubling of the unit cell along *a*, while maintaining hexagonal symmetry. The very low intensity of the superlattice reflections would be compatible with a very slight distortion of the AlPO_4_ framework and/or eventually some slight degree of ordering of the O_2_ guest molecules; however, the number of observed superlattice reflections is too low to constrain such a complicated structural model. A decrease in the *a* lattice parameter of 0.2% is observed at the phase transition, whereas *c* is less affected.

**Figure 6:**
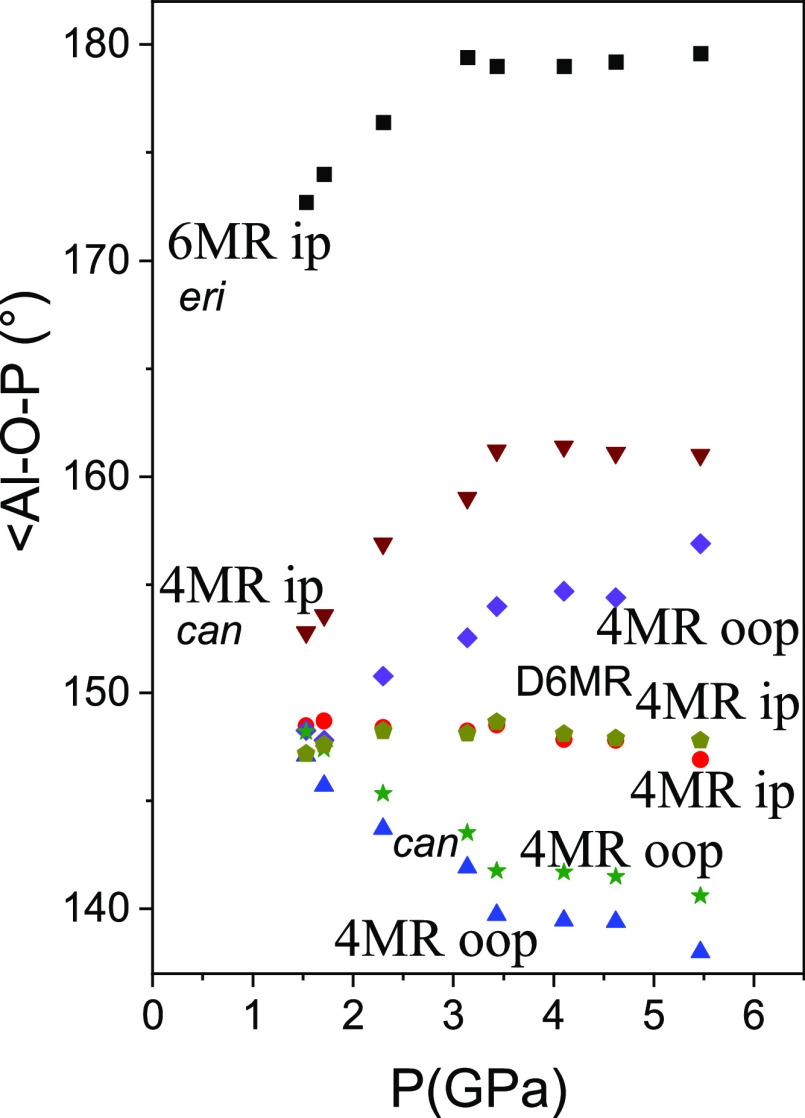
In-plane (ip-*xy*) and out-of-plane (oop) Al-O-P angles in the 4MR and 6MR (see Figure 7) of the O_2_-filled AlPO_4_-17 structure as a function of pressure.

**Figure 7:**
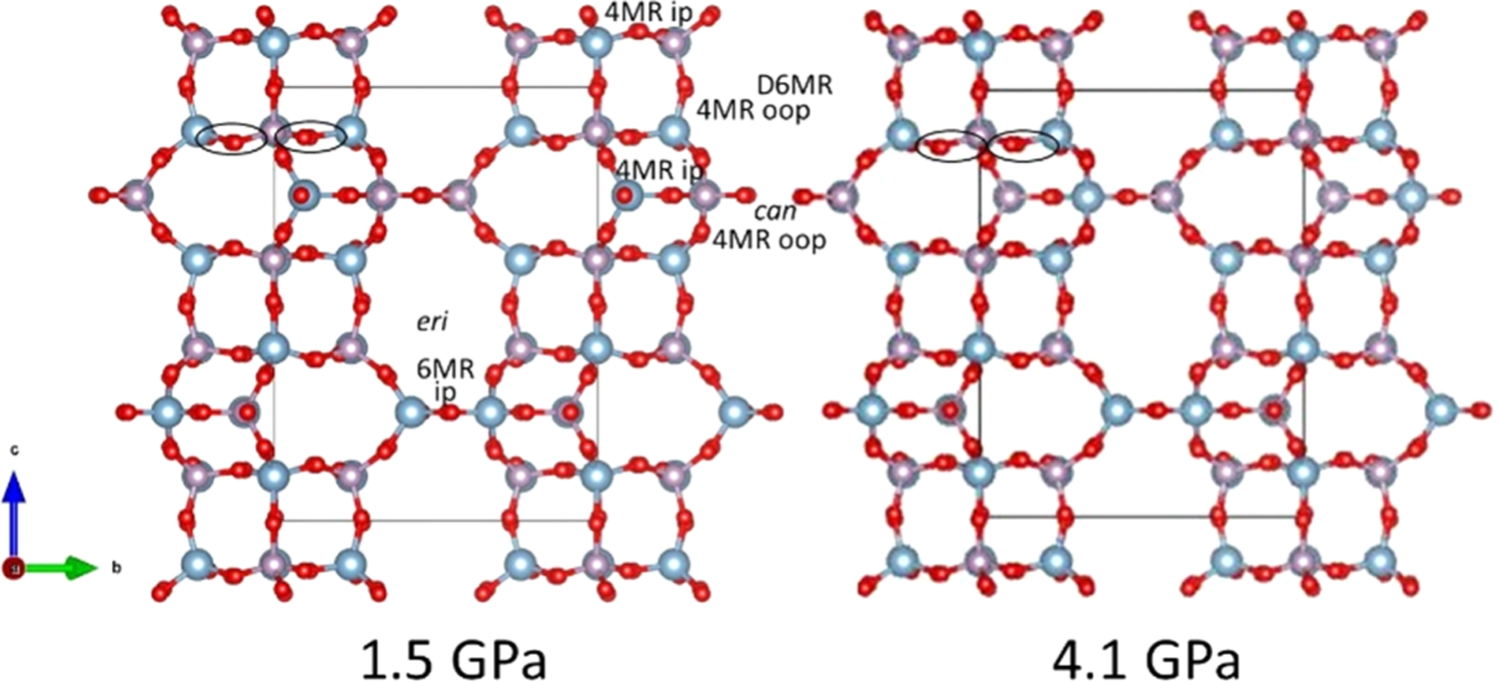
Projections of the crystal structures of O_2_–filled AlPO_4_-17 at 1.5 and 4.1 GPa indicating the in-plane (ip-*xy*) and out-of-plane (oop) Al-O-P angles in the 4MR and 6MR. The ellipses indicate the 4MR ip Al-O-P angles in the D6MR that vary by less than 1° over this pressure interval.

The low compressibility in the *xy* plane is linked to the opening of the in-plane Al-O-P angles in the 6MRs of the erionite cage and the stability of the in-plane Al-O-P angles in the 4MRs belonging to the D6MRs (Figures 6 and 7). The diameter of the 6MRs of the erionite cage is very stable, changing from 2.5052(2) to 2.5025(4) Å from 1.5 to 5.5 GPa. In the case of the D6MRs, the diameter even expands slightly, varying from 2.454(4) to 2.488(5) Å over the same pressure range. The window defined by the 8MR expands in the *xy* plane from 3.31753(15) to 3.5468(5) Å and decreases along the *c* direction from 5.1353(3) to 4.9849(5) Å. There is a flattening of the cancrinite cages along *c* from 0.99696(15) to 0.6863(3) Å corresponding to a decrease in the out-of-plane Al-O-P angles in the 4MRs, decreasing the height of the 8MR window. In contrast, the out-of-plane Al-O-P angles in the 4MRs belonging to the rigid double 6MRs between the cancrinite cages along *c* increase and open the 8MR window in the horizontal plane.

As in many other guest-filled porous materials,^38−41^ in the filled AlPO_4_-17, there is no tendency toward pressure-induced amorphization (PIA), which is essentially complete in the empty form just above 2 GPa. This can be a further indication that the mechanism of PIA is linked to the elastic instability arising from collapse in the *xy* plane, which is suppressed by filling the pores with guests. Molecular dynamics simulation shows that, similarly, the introduction of methane guests in the zeolitic imidazolate framework ZIF-8 increases the C_44_ elastic constant, the decrease in which is linked to the shear instability in the empty form at the origin of pressure-induced amorphization.^42^

## 4 Conclusions

The present results show that argon, nitrogen, and oxygen readily enter the pores of AlPO_4_-17 at high pressure. This results in a pronounced change in the mechanical properties. Almost zero area compressibility is observed in the *xy* plane for AlPO_4_-17 in nitrogen and oxygen, and the elastic instability giving rise to pressure-induced amorphization is suppressed. A phase transition to a structure with a doubled unit cell is observed for AlPO_4_-17 in oxygen. The insertion of nonvolatile guest species could be a method for adapting the mechanical properties of such porous materials, in particular, compressibility and thermal expansion. This guest insertion could be used, for example, to adjust the thermal or elastic response of thin films of these materials to their supports in mechanical, electronic, or optical devices.

## Supporting Information Available

Unit cell parameters as a function of pressure (Tables S1–S3); additional crystallographic results (Figures S1–S3); and full single-crystal data for each pressure for AlPO_4_-17 in O_2_ (Figures S1–S3) (PDF)
