# Supplementary material for: Strongly Modified Mechanical Properties and Phase Transition in AlPO4-17 Due to Insertion of Guest Species at High Pressure
Source: J Phys Chem C Nanomater Interfaces. 2023 Jul 18;127(29):14528–33. doi: 10.1021/acs.jpcc.3c03513 (PMC10389779; doi:10.1021/acs.jpcc.3c03513)
Supplement: Supplementary file 1 — jp3c03513_si_001.pdf [file jp3c03513_si_001.pdf]

Supporting Information - Strongly Modified Mechanical Properties and Phase Transition in $AlPO_4$-17 due to Insertion of Guest Species at High Pressure

Frederico Alabarse‖, Benoît Baptiste ▲, Yoann Guarnelli▲, Boby Joseph ‖, and Julien Haines*†*

‖Elettra Sincrotrone Trieste, Trieste 34149, Italy

▲ Institut de Minéralogie, de Physique des Matériaux et de Cosmochimie, (IMPMC), UMR 7590 CNRS – Sorbonne Université – IRD – MNHN, 4 place Jussieu, 75252 Paris Cedex 5, France.

† Institut Charles Gerhardt Montpellier, CNRS, Université de Montpellier, ENSCM, 34293 Montpellier, France.

AUTHOR INFORMATION

Corresponding Authors

*Frederico G. Alabarse, E: frederico.alabarse@elettra.eu ; T: +39.337.127.0679. ORCID 0000-0002-7375-3666

*Julien Haines, E: julien.haines@umontpellier.fr; T: +33.4.48.79.21.14. ORCID 0000-0002-7030-3213

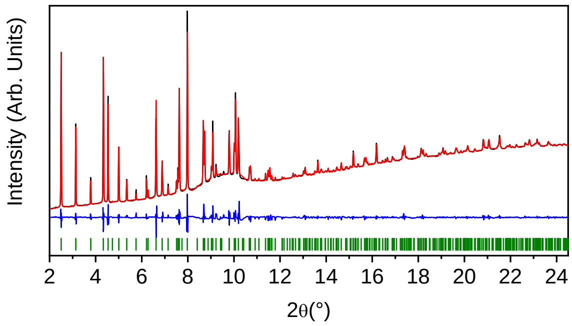

Figure S1. Experimental (black), calculated (red) and difference (blue) profiles (λ=0.4958 Å) from the Le Bail fit (agreement factors Rp: 19.7%, Rwp: 14.3%, Chi^2: 0.141) for nitrogen-filled $AlPO_4$-17 powder at 1.7 GPa. Vertical bars indicate the calculated positions of the Bragg reflections of $AlPO_4$-17. The broad signal near 9.6° is due to fluid N_2.

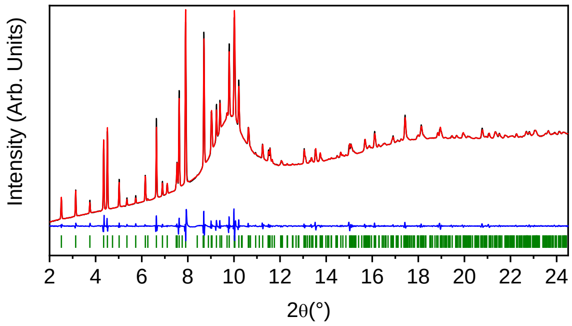

Figure S2. Experimental (black), calculated (red) and difference (blue) profiles (λ=0.4958 Å) from the Le Bail fit (agreement factors Rp: 13.6%, Rwp: 11.9%, Chi^2: 0.094) for argon-filled $AlPO_4$-17 powder at 1.3 GPa. Vertical bars indicate the calculated positions of the Bragg reflections of $AlPO_4$-17. The broad signal near 9.9° is due to fluid Ar.

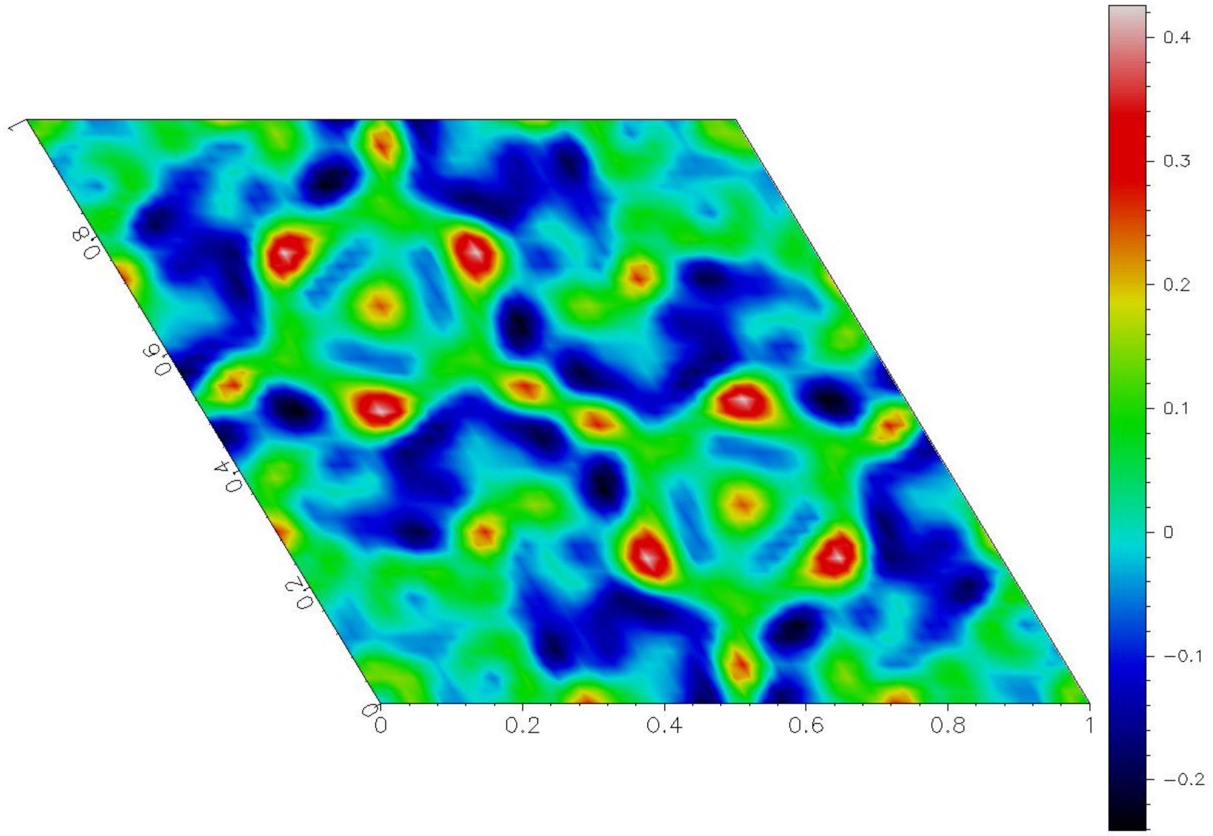

Figure S3. Projection of the Fourier difference map along z for $AlPO_4$-17/O_2 at 1.5 GPa. The scale in e^-/Å^3 is given on the right.

Table S1. Hexagonal unit cell parameters of an $AlPO_4$-17 single crystal in O_2 as a function of pressure. Values in italics correspond to points obtained on decompression, which may be subject to non-equilibrium effects and changes to guest content.

P(GPa)	*a*(Å)	*c*(Å)	*V*(Å^3)
0.1 MPa[1]	13.1113	15.36	2286.65
0.5[2]	13.1181	15.246	2272.0
1.5	13.1028(5)	15.2028(6)	2260.39(15)
1.7	13.0949(9)	15.1539(10)	2250.4(5)
2.3	13.0992(6)	15.1105(7)	2245.4(2)
3.1	13.0853(7)	15.0355(8)	2229.5(2)
3.4	13.0957(6)	15.006(7)	2228.7(2)
4.1	13.0773(6)	14.9450(6)	2213.4(2)
4.6	26.1090(16)	14.9154(7)	8805(1)
5.5	26.0972(18)	14.7950(10)	8726(1)
1.1	*13.0318(5)*	*15.3452(8)*	*2256.89(15)*
0.54	*13.0680(5)*	*15.3384(7)*	*2268.45(16)*
0.43	*13.0695(5)*	*15.3403(6)*	*2269.26(16)*

Table S2. Hexagonal unit cell parameters of $AlPO_4$-17 powder in N_2 as a function of pressure. Values in italics correspond to points obtained on decompression, which may be subject to non-equilibrium effects and changes to guest content.

P(GPa)	*a*(Å)	*c*(Å)	*V*(Å^3)
0.1 MPa[1]	13.1113	15.3600	2286.65
0.53	13.1599(6)	15.1571(6)	2273.3(2)
0.84	13.1570(3)	15.1066(4)	2264.7(1)
1.14	13.1404(3)	15.0813(4)	2255.2(1)
1.46	13.1237(3)	15.0492(4)	2244.7(1)
1.7	13.1104(3)	15.0067(4)	2233.8(1)
2.3	13.2116(4)	14.9017(6)	2252.5(1)
2.6	13.2058(4)	14.8831(7)	2247.8(2)
3.1	13.1917(4)	14.8130(8)	2232.4(2)
3.5	13.1925(5)	14.7579(9)	2224.4(2)
4.0	13.2146(6)	14.662(1)	2217.3(2)
4.5	13.1987(6)	14.624(1)	2206. 3(2)
5.0	13.1776(6)	14.578(1)	2192.2(2)
5.5	13.1636(6)	14.547(1)	2183.0(2)
6.0	13.1572(9)	14.520(1)	2176.9(3)
0.54	*13.1160(6)*	*15.3591(6)*	*2288.2(2)*

Table S3. Hexagonal unit cell parameters of $AlPO_4$-17 powder in Ar as a function of pressure.

P(GPa)	*a*(Å)	*c*(Å)	*V*(Å^3)
0.1 MPa[1]	13.1113	15.36	2286.65
0.33	13.1456(9)	15.1984(9)	2274.5(2)
0.73	13.1332(4)	15.1725(5)	2266.3(1)
0.98	13.1153(5)	15.1846(6)	2262.0(1)
1.31	13.0921(5)	15.1824(6)	2253.7(2)
1.68	13.2374(3)	14.8007(4)	2246.1(1)
2.7	13.2261(4)	14.6647(4)	2221.6(1)
3.6	13.1758(4)	14.5909(4)	2193.7(1)
5.0	13.144(1)	14.386(1)	2152.4(3)
6.0	13.061(6)	14.208(1)	2099.8(2)

References

1. Alabarse, F. G.; Silly, G.; Brubach, J. B.; Roy, P.; Haidoux, A.; Levelut, C.; Bantignies, J. L.; Kohara, S.; Le Floch, S.; Cambon, O.; Haines, J. Anomalous Compressibility and Amorphization in $AlPO_4$-17, the Oxide with the Highest Negative Thermal Expansion. *J. Phys. Chem. C* **2017,** *121* (12), 6852-6863.

2. Alabarse, F.; Baptiste, B.; Joseph, B.; Haines, J. Tuning Negative Thermal Expansion in $AlPO_4$-17 by Insertion of Guest Molecules. *J. Phys. Chem. Lett.* **2022,** *13*, 9390-9395.

X-ray crystallographic data

$AlPO_4$-17/O_2 0.54GPa

Table 1 Crystal data and structure refinement for 17O2_sc2_0p54GPa.

Identification code	17O2_sc2_0p54GPa
Empirical formula	AlO_4P
Formula weight	121.95
Temperature/K	293(2)
Crystal system	hexagonal
Space group	$P6_3/m$
a/Å	13.0680(5)
b/Å	13.0680(5)
c/Å	15.3384(7)
α/°	90
β/°	90
γ/°	120
Volume/Å^3	2268.4(2)
Z	18
ρ_{calc}g/cm^3	1.607
μ/mm^{-1}	0.223
F(000)	1080.0
Crystal size/mm^3	0.125 × 0.050 × 0.050
Radiation	synchrotron (λ = 0.49555)
2Θ range for data collection/°	4.346 to 31.89
Index ranges	$-11 \le h \le 11$, $-11 \le k \le 10$, $-16 \le l \le 16$
Reflections collected	3106
Independent reflections	1073 [R_{int} = 0.0250, R_{sigma} = 0.0309]
Data/restraints/parameters	1073/0/88
Goodness-of-fit on F^2	1.078
Final R indexes [I>=2σ (I)]	R_1 = 0.0284, wR_2 = 0.0825
Final R indexes [all data]	R_1 = 0.0375, wR_2 = 0.0856
Largest diff. peak/hole / e Å^{-3}	0.25/-0.25

Table 2 Fractional Atomic Coordinates (×10^4) and Equivalent Isotropic Displacement Parameters (Å^2×10^3) for 17O2_sc2_0p54GPa. U_{eq} is defined as 1/3 of the trace of the orthogonalised U_{IJ} tensor.

Atom	*x*	*y*	*z*	U(eq)
P1	6671.3(8)	10923.1(9)	7500	21.9(3)
P2	7598.8(7)	7625.4(6)	6007.0(4)	24.2(3)
Al2	6690.7(9)	5765.5(10)	7500	21.3(3)

Table 2 Fractional Atomic Coordinates (×10^4) and Equivalent Isotropic Displacement Parameters (Å^2×10^3) for 17O2_sc2_0p54GPa. U_{eq} is defined as 1/3 of the trace of the orthogonalised U_{IJ} tensor.

Atom	*x*	*y*	*z*	U(eq)
Al1	7631.3(7)	10026.0(7)	6000.3(5)	23.7(3)
O1	5467(2)	10809(2)	7500	30.0(7)
O4	7455.9(17)	8670.6(16)	6255.3(12)	35.2(5)
O5	6835.3(18)	6594.3(16)	6586.1(11)	35.2(5)
O2	7614(2)	12225(2)	7500	37.9(8)
O6	7210.0(19)	7256.9(19)	5069.4(11)	38.2(5)
O3	6786.9(18)	10316.5(19)	6695.0(11)	37.7(6)
O7	8892.8(18)	7984.7(19)	6116.1(13)	42.4(6)

Table 3 Anisotropic Displacement Parameters (Å^2×10^3) for 17O2_sc2_0p54GPa. The Anisotropic displacement factor exponent takes the form: -2π^2[h^{2}a*^{2}U$_{11}$+2hka*b*U$_{12}$+…].

Atom	U_{11}	U_{22}	U_{33}	U_{23}	U_{13}	U_{12}
P1	17.7(6)	20.7(6)	28.6(5)	0	0	10.7(5)
P2	25.2(5)	21.9(5)	27.5(5)	3.2(3)	2.7(3)	13.3(4)
Al2	17.3(7)	16.7(7)	26.6(6)	0	0	6.0(6)
Al1	25.1(5)	21.0(5)	25.6(5)	-0.4(3)	2.2(4)	12.0(4)
O1	21.2(16)	32.7(17)	37.9(15)	0	0	14.9(14)
O4	41.1(13)	25.9(12)	44.0(11)	3.9(9)	6.2(10)	20.8(11)
O5	39.9(13)	25.5(12)	35.2(11)	7.8(8)	3.0(9)	12.5(11)
O2	25.9(17)	27.9(17)	57.1(18)	0	0	11.4(15)
O6	41.7(13)	39.7(13)	29.1(11)	-1.2(9)	-2.7(9)	17.3(11)
O3	37.8(14)	42.6(13)	39.2(12)	-5.5(10)	2.9(9)	25.1(11)
O7	30.2(13)	41.7(14)	59.4(14)	-4.6(11)	-2.4(10)	21.0(11)

Table 4 Bond Lengths for 17O2_sc2_0p54GPa.

Atom	Atom	Length/Å	Atom	Atom	Length/Å
P1	O1	1.504(3)	Al2	O5^1	1.7232(18)
P1	O2	1.522(3)	Al2	O2^3	1.716(3)
P1	O3	1.5151(19)	Al1	O4	1.7137(19)
P1	O3^1	1.5151(19)	Al1	O6^4	1.7163(19)
P2	O4	1.5168(19)	Al1	O3	1.7077(19)
P2	O5	1.5019(19)	Al1	O7^5	1.725(2)
P2	O6	1.5210(18)	O1	Al2^6	1.692(3)
P2	O7	1.521(2)	O2	Al2^5	1.716(3)
Al2	O1^2	1.692(3)	O6	Al1^7	1.7163(19)
Al2	O5	1.7232(18)	O7	Al1^3	1.725(2)

[1]+X,+Y,3/2-Z; [2]+Y-X,1-X,+Z; [3]2-Y,1+X-Y,+Z; [4]+Y,1-X+Y,1-Z; [5]1+Y-X,2-X,+Z; [6]1-Y,1+X-Y,+Z; [7]1-Y+X,+X,1-Z

Table 5 Bond Angles for 17O2_sc2_0p54GPa.

Atom	Atom	Atom	Angle/°	Atom	Atom	Atom	Angle/°
O1	P1	O2	109.46(16)	O2[3]	Al2	O5	109.47(10)
O1	P1	O3[1]	108.72(10)	O2[3]	Al2	O5[1]	109.47(10)
O1	P1	O3	108.72(10)	O4	Al1	O6[4]	110.05(10)
O3[1]	P1	O2	110.37(11)	O4	Al1	O7[5]	110.16(11)
O3	P1	O2	110.37(11)	O6[4]	Al1	O7[5]	107.04(11)
O3[1]	P1	O3	109.16(17)	O3	Al1	O4	108.66(10)
O4	P2	O6	110.52(11)	O3	Al1	O6[4]	111.84(11)
O4	P2	O7	108.22(12)	O3	Al1	O7[5]	109.09(11)
O5	P2	O4	109.49(11)	P1	O1	Al2[6]	170.60(19)
O5	P2	O6	107.95(12)	P2	O4	Al1	149.35(13)
O5	P2	O7	110.44(12)	P2	O5	Al2	149.31(14)
O6	P2	O7	110.21(12)	P1	O2	Al2[5]	150.2(2)
O1[2]	Al2	O5	109.45(9)	P2	O6	Al1[7]	143.93(16)
O1[2]	Al2	O5[1]	109.45(9)	P1	O3	Al1	150.39(15)
O1[2]	Al2	O2[3]	110.10(15)	P2	O7	Al1[3]	147.57(15)
O5	Al2	O5[1]	108.89(14)				

[1]+X,+Y,3/2-Z; [2]+Y-X,1-X,+Z; [3]2-Y,1+X-Y,+Z; [4]+Y,1-X+Y,1-Z; [5]1+Y-X,2-X,+Z; [6]1-Y,1+X-Y,+Z; [7]1-Y+X,+X,1-Z

Table 6 Torsion Angles for 17O2_sc2_0p54GPa.

A	B	C	D	Angle/°	A	B	C	D	Angle/°
O1	P1	O2	Al2[1]	180.0	O6	P2	O5	Al2	143.6(3)
O1	P1	O3	Al1	168.7(3)	O6	P2	O7	Al1[4]	-55.1(3)
O1[2]	Al2	O5	P2	-171.8(3)	O6[6]	Al1	O4	P2	41.9(3)
O4	P2	O5	Al2	-96.0(3)	O6[6]	Al1	O3	P1	-138.5(3)
O4	P2	O6	Al1[3]	74.8(3)	O3[5]	P1	O2	Al2[1]	60.37(10)
O4	P2	O7	Al1[4]	-176.1(2)	O3	P1	O2	Al2[1]	-60.37(10)
O4	Al1	O3	P1	99.8(3)	O3[5]	P1	O3	Al1	-72.8(4)
O5	P2	O4	Al1	-163.5(3)	O3	Al1	O4	P2	164.7(3)
O5	P2	O6	Al1[3]	-165.5(2)	O7	P2	O4	Al1	76.1(3)
O5	P2	O7	Al1[4]	64.1(3)	O7	P2	O5	Al2	23.0(3)
O5[5]	Al2	O5	P2	68.6(3)	O7	P2	O6	Al1[3]	-44.8(3)
O2	P1	O3	Al1	48.6(3)	O7[1]	Al1	O4	P2	-75.9(3)
O2[4]	Al2	O5	P2	-51.0(3)	O7[1]	Al1	O3	P1	-20.3(3)
O6	P2	O4	Al1	-44.7(3)					

[1]1+Y-X,2-X,+Z; [2]+Y-X,1-X,+Z; [3]1-Y+X,+X,1-Z; [4]2-Y,1+X-Y,+Z; [5]+X,+Y,3/2-Z; [6]+Y,1-X+Y,1-Z

Table 7 Solvent masks information for 17O2_sc2_0p54GPa.

Number	X	Y	Z	Volume	Electron count	Content
1	0.000	0.000	0.000	9.1	0.0	?
2	0.000	0.000	0.250	25.0	21.0	?
3	0.000	0.000	0.500	9.1	0.0	?
4	0.000	0.000	0.750	25.0	21.0	?
5	-0.136	-0.208	0.067	899.5	212.3	?

Experimental

Single crystals of AlO_4P **[17O2_sc2_0p54GPa]** were **[]**. A suitable crystal was selected and **[]** on a **esperanto-CrysAlisPro-abstract goniometer imported esperanto images** diffractometer. The crystal was kept at 293(2) K during data collection. Using Olex2 [1], the structure was solved with the SHELXT [2] structure solution program using Intrinsic Phasing and refined with the SHELXL [3] refinement package using Least Squares minimisation.

1. Dolomanov, O.V., Bourhis, L.J., Gildea, R.J, Howard, J.A.K. & Puschmann, H. (2009), J. Appl. Cryst. 42, 339-341.
2. Sheldrick, G.M. (2015). Acta Cryst. A71, 3-8.
3. Sheldrick, G.M. (2015). Acta Cryst. C71, 3-8.

Crystal structure determination of [17O2_sc2_0p54GPa]

Crystal Data for AlO_4P (M =121.95 g/mol): hexagonal, space group P6$_3$/m (no. 176), a = 13.0680(5) Å, c = 15.3384(7) Å, V = 2268.4(2) Å^3, Z = 18, T = 293(2) K, μ(synchrotron) = 0.223 mm^{-1}, $Dcalc$ = 1.607 g/cm^3, 3106 reflections measured (4.346° ≤ 2Θ ≤ 31.89°), 1073 unique (R_{int} = 0.0250, R_{sigma} = 0.0309) which were used in all calculations. The final R_1 was 0.0284 (I > 2σ(I)) and wR_2 was 0.0856 (all data).

Refinement model description

Number of restraints - 0, number of constraints - unknown.
Details:
N/A

This report has been created with Olex2, compiled on 2023.03.06 svn.rbb2c1857 for OlexSys. Please let us know if there are any errors or if you would like to have additional features

$AlPO_4$-17/O_2 1.10GPa

Table 1 Crystal data and structure refinement for 17O2_sc2_1p10GPa.

Identification code	17O2_sc2_1p10GPa
Empirical formula	AlO_4P
Formula weight	121.95
Temperature/K	293(2)
Crystal system	hexagonal
Space group	$P6_3/m$
a/Å	13.0318(5)
b/Å	13.0318(5)
c/Å	15.3452(6)
α/°	90
β/°	90
γ/°	120
Volume/Å^3	2256.90(19)
Z	18
ρ_{calc}g/cm^3	1.615
μ/mm^{-1}	0.224
F(000)	1080.0
Crystal size/mm^3	0.125 × 0.050 × 0.050
Radiation	synchrotron (λ = 0.49555)
2Θ range for data collection/°	4.358 to 31.984
Index ranges	$-11 \leq h \leq 12$, $-11 \leq k \leq 11$, $-16 \leq l \leq 16$
Reflections collected	3502
Independent reflections	1106 [R_{int} = 0.0279, R_{sigma} = 0.0328]
Data/restraints/parameters	1106/0/88
Goodness-of-fit on F^2	1.081
Final R indexes [I>=2σ (I)]	R_1 = 0.0316, wR_2 = 0.0890
Final R indexes [all data]	R_1 = 0.0418, wR_2 = 0.0919
Largest diff. peak/hole / e Å^{-3}	0.24/-0.25

Table 2 Fractional Atomic Coordinates (×10^4) and Equivalent Isotropic Displacement Parameters (Å^2×10^3) for 17O2_sc2_1p10GPa. U_{eq} is defined as 1/3 of the trace of the orthogonalised U_{IJ} tensor.

Atom	*x*	*y*	*z*	U(eq)
P1	6668.3(9)	10918.7(10)	7500	22.7(3)
P2	7596.4(7)	7621.5(7)	6002.1(5)	25.0(3)
Al2	6689.7(10)	5767.9(10)	7500	22.2(4)
Al1	7626.1(8)	10024.8(7)	5996.1(5)	24.4(3)
O1	5464(2)	10801(2)	7500	31.7(7)

Table 2 Fractional Atomic Coordinates (×10^4) and Equivalent Isotropic Displacement Parameters (Å^2×10^3) for 17O2_sc2_1p10GPa. U_{eq} is defined as 1/3 of the trace of the orthogonalised U_{IJ} tensor.

Atom	*x*	*y*	*z*	U(eq)
O4	7456.4(18)	8670.4(17)	6249.3(13)	35.8(6)
O5	6835(2)	6593.5(18)	6585.1(12)	38.6(6)
O2	7608(3)	12217(3)	7500	39.7(8)
O6	7201(2)	7244(2)	5068.5(11)	39.4(6)
O3	6786(2)	10317(2)	6696.6(11)	40.1(6)
O7	8891.0(18)	7984(2)	6103.9(14)	43.0(6)

Table 3 Anisotropic Displacement Parameters (Å^2×10^3) for 17O2_sc2_1p10GPa. The Anisotropic displacement factor exponent takes the form: $-2\pi^2[h^2a^{*2}U_{11}+2hka^*b^*U_{12}+\ldots]$.

Atom	U_{11}	U_{22}	U_{33}	U_{23}	U_{13}	U_{12}
P1	19.4(7)	23.0(7)	27.7(6)	0	0	12.2(6)
P2	27.0(5)	24.7(5)	25.9(5)	2.7(3)	2.7(3)	14.9(4)
Al2	19.2(8)	17.1(7)	26.7(7)	0	0	6.5(6)
Al1	26.3(6)	22.0(6)	24.6(5)	-0.5(4)	2.0(4)	12.0(4)
O1	23.3(18)	34.9(18)	39.2(16)	0	0	16.3(15)
O4	38.4(14)	27.2(13)	45.1(11)	5.2(10)	7.4(10)	19.0(11)
O5	47.6(15)	29.7(14)	34.3(11)	8.9(9)	4.0(10)	16.0(12)
O2	23.3(18)	27.6(18)	59.7(19)	0	0	6.4(16)
O6	42.0(14)	42.0(14)	32.0(12)	-1.6(9)	-2.0(9)	19.4(12)
O3	44.8(15)	46.6(15)	38.3(12)	-4.0(10)	5.0(10)	29.9(13)
O7	30.7(14)	42.0(14)	58.3(15)	-4.6(11)	-2.1(11)	19.7(12)

Table 4 Bond Lengths for 17O2_sc2_1p10GPa.

Atom	Atom	Length/Å	Atom	Atom	Length/Å
P1	O1	1.498(3)	Al2	O5^1	1.7207(19)
P1	O2	1.514(3)	Al2	O2^3	1.724(3)
P1	O3^1	1.5095(19)	Al1	O4	1.710(2)
P1	O3	1.5095(19)	Al1	O6^4	1.7116(19)
P2	O4	1.515(2)	Al1	O3	1.708(2)
P2	O5	1.500(2)	Al1	O7^5	1.725(2)
P2	O6	1.5189(19)	O1	Al2^6	1.690(3)
P2	O7	1.516(2)	O2	Al2^5	1.724(3)
Al2	O1^2	1.690(3)	O6	Al1^7	1.7115(19)
Al2	O5	1.7207(19)	O7	Al1^3	1.725(2)

^{1}+X,+Y,3/2-Z; ^{2}+Y-X,1-X,+Z; ^{3}2-Y,1+X-Y,+Z; ^{4}+Y,1-X+Y,1-Z; ^{5}1+Y-X,2-X,+Z; ^{6}1-Y,1+X-Y,+Z; ^{7}1-Y+X,+X,1-Z

Table 5 Bond Angles for 17O2_sc2_1p10GPa.

Atom	Atom	Atom	Angle/°	Atom	Atom	Atom	Angle/°
O1	P1	O2	109.57(17)	O5	Al2	O2[3]	109.27(11)
O1	P1	O3[1]	108.66(11)	O5[1]	Al2	O2[3]	109.27(11)
O1	P1	O3	108.66(11)	O4	Al1	O6[4]	110.03(11)
O3[1]	P1	O2	110.20(12)	O4	Al1	O7[5]	110.00(11)
O3	P1	O2	110.20(12)	O6[4]	Al1	O7[5]	106.94(11)
O3[1]	P1	O3	109.51(18)	O3	Al1	O4	108.70(11)
O4	P2	O6	110.76(12)	O3	Al1	O6[4]	111.97(12)
O4	P2	O7	108.09(13)	O3	Al1	O7[5]	109.18(11)
O5	P2	O4	109.39(12)	P1	O1	Al2[6]	170.3(2)
O5	P2	O6	107.89(13)	P2	O4	Al1	149.60(14)
O5	P2	O7	110.68(13)	P2	O5	Al2	149.51(16)
O7	P2	O6	110.03(12)	P1	O2	Al2[5]	150.2(2)
O1[2]	Al2	O5	109.29(10)	P2	O6	Al1[7]	143.25(17)
O1[2]	Al2	O5[1]	109.29(10)	P1	O3	Al1	150.78(16)
O1[2]	Al2	O2[3]	110.33(15)	P2	O7	Al1[3]	147.67(16)
O5	Al2	O5[1]	109.36(15)				

[1]+X,+Y,3/2-Z; [2]+Y-X,1-X,+Z; [3]2-Y,1+X-Y,+Z; [4]+Y,1-X+Y,1-Z; [5]1+Y-X,2-X,+Z; [6]1-Y,1+X-Y,+Z; [7]1-Y+X,+X,1-Z

Table 6 Torsion Angles for 17O2_sc2_1p10GPa.

A	B	C	D	Angle/°	A	B	C	D	Angle/°
O1	P1	O2	Al2[1]	180.000(0)	O6	P2	O5	Al2	143.6(3)
O1	P1	O3	Al1	168.7(3)	O6	P2	O7	Al1[4]	-56.2(3)
O1[2]	Al2	O5	P2	-171.7(3)	O6[6]	Al1	O4	P2	41.7(3)
O4	P2	O5	Al2	-95.8(3)	O6[6]	Al1	O3	P1	-138.7(3)
O4	P2	O6	Al1[3]	74.3(3)	O3[5]	P1	O2	Al2[1]	60.49(11)
O4	P2	O7	Al1[4]	-177.3(2)	O3	P1	O2	Al2[1]	-60.49(11)
O4	Al1	O3	P1	99.5(3)	O3[5]	P1	O3	Al1	-72.8(4)
O5	P2	O4	Al1	-163.3(3)	O3	Al1	O4	P2	164.6(3)
O5	P2	O6	Al1[3]	-166.0(2)	O7	P2	O4	Al1	76.1(3)
O5	P2	O7	Al1[4]	62.9(3)	O7	P2	O5	Al2	23.2(3)
O5[5]	Al2	O5	P2	68.7(4)	O7	P2	O6	Al1[3]	-45.1(3)
O2	P1	O3	Al1	48.6(4)	O7[1]	Al1	O4	P2	-75.9(3)
O2[4]	Al2	O5	P2	-50.9(3)	O7[1]	Al1	O3	P1	-20.5(4)
O6	P2	O4	Al1	-44.5(3)					

[1]1+Y-X,2-X,+Z; [2]+Y-X,1-X,+Z; [3]1-Y+X,+X,1-Z; [4]2-Y,1+X-Y,+Z; [5]+X,+Y,3/2-Z; [6]+Y,1-X+Y,1-Z

Table 7 Solvent masks information for 17O2_sc2_1p10GPa.

Number	X	Y	Z	Volume	Electron count	Content
1	0.000	0.000	0.000	7.2	2.1	?
2	0.000	0.000	0.250	26.1	27.6	?
3	0.000	0.000	0.500	7.2	2.1	?
4	0.000	0.000	0.750	26.1	27.6	?
5	-0.352	0.114	-0.226	892.8	263.6	?

Experimental

Single crystals of AlO_4P **[17O2_sc2_1p10GPa]** were **[]**. A suitable crystal was selected and **[]** on a **esperanto-CrysAlisPro-abstract goniometer imported esperanto images** diffractometer. The crystal was kept at 293(2) K during data collection. Using Olex2 [1], the structure was solved with the SHELXT [2] structure solution program using Intrinsic Phasing and refined with the SHELXL [3] refinement package using Least Squares minimisation.

1. Dolomanov, O.V., Bourhis, L.J., Gildea, R.J, Howard, J.A.K. & Puschmann, H. (2009), J. Appl. Cryst. 42, 339-341.
2. Sheldrick, G.M. (2015). Acta Cryst. A71, 3-8.
3. Sheldrick, G.M. (2015). Acta Cryst. C71, 3-8.

Crystal structure determination of [17O2_sc2_1p10GPa]

Crystal Data for AlO_4P (M =121.95 g/mol): hexagonal, space group $P6_3/m$ (no. 176), a = 13.0318(5) Å, c = 15.3452(6) Å, V = 2256.90(19) $Å^3$, Z = 18, T = 293(2) K, μ(synchrotron) = 0.224 mm^{-1}, $Dcalc$ = 1.615 g/cm^3, 3502 reflections measured (4.358° ≤ 2Θ ≤ 31.984°), 1106 unique (R_{int} = 0.0279, R_{sigma} = 0.0328) which were used in all calculations. The final R_1 was 0.0316 (I > 2σ(I)) and wR_2 was 0.0919 (all data).

Refinement model description

Number of restraints - 0, number of constraints - unknown.
Details:
N/A

This report has been created with Olex2, compiled on 2023.03.06 svn.rbb2c1857 for OlexSys. Please let us know if there are any errors or if you would like to have additional features.

$AlPO_4$-17/O_2 1.53GPa

Table 1 Crystal data and structure refinement for 17O2_sc2_1p53GPa.

Identification code	17O2_sc2_1p53GPa
Empirical formula	AlO_4P
Formula weight	121.95
Temperature/K	293(2)
Crystal system	hexagonal
Space group	$P6_3/m$
a/Å	13.1028(5)
b/Å	13.1028(5)
c/Å	15.2028(6)
α/°	90
β/°	90
γ/°	120
Volume/Å^3	2260.38(19)
Z	18
ρ_{calc}g/cm^3	1.613
μ/mm^{-1}	0.224
F(000)	1080.0
Crystal size/mm^3	0.125 × 0.050 × 0.050
Radiation	synchrotron (λ = 0.49555)
2Θ range for data collection/°	4.334 to 31.934
Index ranges	$-11 \leq h \leq 11$, $-11 \leq k \leq 10$, $-16 \leq l \leq 16$
Reflections collected	3045
Independent reflections	1063 [R_{int} = 0.0348, R_{sigma} = 0.0448]
Data/restraints/parameters	1063/0/88
Goodness-of-fit on F^2	1.086
Final R indexes [I>=2σ (I)]	R_1 = 0.0335, wR_2 = 0.0958
Final R indexes [all data]	R_1 = 0.0425, wR_2 = 0.0987
Largest diff. peak/hole / e Å^{-3}	0.24/-0.32

Table 2 Fractional Atomic Coordinates (×10^4) and Equivalent Isotropic Displacement Parameters (Å^2×10^3) for 17O2_sc2_1p53GPa. U_{eq} is defined as 1/3 of the trace of the orthogonalised U_{IJ} tensor.

Atom	*x*	*y*	*z*	U(eq)
P1	6666.3(10)	10920.6(10)	7500	23.4(4)
P2	7617.3(7)	7640.5(7)	6024.2(5)	24.7(3)
Al2	6691.3(11)	5762.1(11)	7500	23.3(4)
Al1	7642.9(8)	10023.8(8)	6016.9(6)	24.5(3)
O1	5486(2)	10835(3)	7500	29.2(8)

Table 2 Fractional Atomic Coordinates (×10[4]) and Equivalent Isotropic Displacement Parameters (Å[2]×10[3]) for 17O2_sc2_1p53GPa. U_{eq} is defined as 1/3 of the trace of the orthogonalised U_{IJ} tensor.

Atom	*x*	*y*	*z*	U(eq)
O4	7476(2)	8676.9(19)	6284.1(13)	34.4(6)
O5	6824(2)	6586.9(18)	6583.8(13)	36.4(6)
O2	7620(3)	12200(3)	7500	40.1(9)
O6	7277(2)	7312(2)	5071.1(12)	38.5(6)
O3	6769(2)	10314(2)	6689.7(13)	38.0(6)
O7	8900(2)	7985(2)	6161.0(15)	42.7(7)

Table 3 Anisotropic Displacement Parameters (Å[2]×10[3]) for 17O2_sc2_1p53GPa. The Anisotropic displacement factor exponent takes the form: -$2\pi^2[h^2a^{*2}U_{11}+2hka^*b^*U_{12}+\ldots]$.

Atom	U_{11}	U_{22}	U_{33}	U_{23}	U_{13}	U_{12}
P1	20.3(7)	24.6(7)	28.3(6)	0	0	13.5(6)
P2	25.8(5)	23.7(5)	26.5(5)	2.6(4)	2.3(4)	13.8(4)
Al2	20.8(8)	19.8(8)	26.4(7)	0	0	8.0(7)
Al1	26.1(6)	22.1(6)	25.2(6)	-0.1(4)	1.7(4)	12.0(5)
O1	24.9(19)	29.4(19)	34.9(16)	0	0	14.7(16)
O4	42.0(16)	27.6(14)	39.4(12)	2.4(10)	4.2(10)	21.8(13)
O5	44.9(15)	29.4(14)	32.7(12)	8.4(10)	6.5(10)	16.8(13)
O2	23(2)	25(2)	64(2)	0	0	5.7(17)
O6	44.6(15)	44.9(16)	27.2(12)	0.5(10)	-2.2(10)	23.3(13)
O3	42.6(16)	44.7(15)	33.9(13)	-5.0(11)	3.5(11)	27.2(13)
O7	30.6(15)	42.8(16)	58.2(16)	-7.6(12)	-2.6(11)	20.9(13)

Table 4 Bond Lengths for 17O2_sc2_1p53GPa.

Atom	Atom	Length/Å	Atom	Atom	Length/Å
P1	O1	1.494(3)	Al2	O5[1]	1.718(2)
P1	O2	1.509(3)	Al2	O2[3]	1.721(3)
P1	O3	1.508(2)	Al1	O4	1.715(2)
P1	O3[1]	1.508(2)	Al1	O6[4]	1.712(2)
P2	O4	1.512(2)	Al1	O3	1.714(2)
P2	O5	1.508(2)	Al1	O7[5]	1.715(2)
P2	O6	1.514(2)	O1	Al2[6]	1.700(3)
P2	O7	1.521(2)	O2	Al2[5]	1.721(3)
Al2	O1[2]	1.700(3)	O6	Al1[7]	1.712(2)
Al2	O5	1.718(2)	O7	Al1[3]	1.715(2)

[1]+X,+Y,3/2-Z; [2]+Y-X,1-X,+Z; [3]2-Y,1+X-Y,+Z; [4]+Y,1-X+Y,1-Z; [5]1+Y-X,2-X,+Z; [6]1-Y,1+X-Y,+Z; [7]1-Y+X,+X,1-Z

Table 5 Bond Angles for 17O2_sc2_1p53GPa.

Atom	Atom	Atom	Angle/°	Atom	Atom	Atom	Angle/°
O1	P1	O2	109.55(18)	O5	Al2	O2[3]	109.32(11)
O1	P1	O3	108.82(11)	O5[1]	Al2	O2[3]	109.33(11)
O1	P1	O3[1]	108.82(11)	O6[4]	Al1	O4	109.57(11)
O3	P1	O2	110.06(12)	O6[4]	Al1	O3	112.01(12)
O3[1]	P1	O2	110.06(12)	O6[4]	Al1	O7[5]	107.22(12)
O3	P1	O3[1]	109.50(19)	O3	Al1	O4	109.32(12)
O4	P2	O6	110.77(12)	O3	Al1	O7[5]	108.98(12)
O4	P2	O7	108.14(14)	O7[5]	Al1	O4	109.70(12)
O5	P2	O4	109.66(13)	P1	O1	Al2[6]	172.7(2)
O5	P2	O6	108.02(14)	P2	O4	Al1	148.47(15)
O5	P2	O7	110.39(13)	P2	O5	Al2	147.10(16)
O6	P2	O7	109.86(14)	P1	O2	Al2[5]	152.8(2)
O1[2]	Al2	O5	109.61(10)	P2	O6	Al1[7]	148.25(19)
O1[2]	Al2	O5[1]	109.61(10)	P1	O3	Al1	148.21(16)
O1[2]	Al2	O2[3]	110.57(16)	P2	O7	Al1[3]	147.18(17)
O5	Al2	O5[1]	108.36(16)				

[1]+X,+Y,3/2-Z; [2]+Y-X,1-X,+Z; [3]2-Y,1+X-Y,+Z; [4]+Y,1-X+Y,1-Z; [5]1+Y-X,2-X,+Z; [6]1-Y,1+X-Y,+Z; [7]1-Y+X,+X,1-Z

Table 6 Torsion Angles for 17O2_sc2_1p53GPa.

A	B	C	D	Angle/°	A	B	C	D	Angle/°
O1	P1	O2	Al2[1]	180.0	O6	P2	O5	Al2	143.4(3)
O1	P1	O3	Al1	169.8(3)	O6	P2	O7	Al1[4]	-51.0(3)
O1[2]	Al2	O5	P2	-172.0(3)	O6[6]	Al1	O4	P2	38.6(3)
O4	P2	O5	Al2	-95.8(3)	O6[6]	Al1	O3	P1	-140.4(3)
O4	P2	O6	Al1[3]	73.2(3)	O3	P1	O2	Al2[1]	-60.39(11)
O4	P2	O7	Al1[4]	-172.0(3)	O3[5]	P1	O2	Al2[1]	60.39(11)
O4	Al1	O3	P1	98.0(3)	O3[5]	P1	O3	Al1	-71.3(4)
O5	P2	O4	Al1	-160.3(3)	O3	Al1	O4	P2	161.7(3)
O5	P2	O6	Al1[3]	-166.7(3)	O7	P2	O4	Al1	79.3(3)
O5	P2	O7	Al1[4]	68.1(3)	O7	P2	O5	Al2	23.3(3)
O5[5]	Al2	O5	P2	68.4(4)	O7	P2	O6	Al1[3]	-46.2(3)
O2	P1	O3	Al1	49.8(4)	O7[1]	Al1	O4	P2	-78.8(3)
O2[4]	Al2	O5	P2	-50.6(3)	O7[1]	Al1	O3	P1	-21.9(3)
O6	P2	O4	Al1	-41.1(4)					

[1]1+Y-X,2-X,+Z; [2]+Y-X,1-X,+Z; [3]1-Y+X,+X,1-Z; [4]2-Y,1+X-Y,+Z; [5]+X,+Y,3/2-Z; [6]+Y,1-X+Y,1-Z

Table 7 Solvent masks information for 17O2_sc2_1p53GPa.

Number	X	Y	Z	Volume	Electron count	Content
1	0.000	0.000	0.000	11.0	2.1	?
2	0.000	0.000	0.250	20.5	25.9	?
3	0.000	0.000	0.500	11.0	2.1	?
4	0.000	0.000	0.750	20.5	25.9	?
5	-0.940	-0.700	-0.016	899.1	309.3	?

Experimental

Single crystals of AlO_4P **[17O2_sc2_1p53GPa]** were **[]**. A suitable crystal was selected and **[]** on a **esperanto-CrysAlisPro-abstract goniometer imported esperanto images** diffractometer. The crystal was kept at 293(2) K during data collection. Using Olex2 [1], the structure was solved with the SHELXT [2] structure solution program using Intrinsic Phasing and refined with the SHELXL [3] refinement package using Least Squares minimisation.

1. Dolomanov, O.V., Bourhis, L.J., Gildea, R.J, Howard, J.A.K. & Puschmann, H. (2009), J. Appl. Cryst. 42, 339-341.
2. Sheldrick, G.M. (2015). Acta Cryst. A71, 3-8.
3. Sheldrick, G.M. (2015). Acta Cryst. C71, 3-8.

Crystal structure determination of [17O2_sc2_1p53GPa]

Crystal Data for AlO_4P (M =121.95 g/mol): hexagonal, space group $P6_3/m$ (no. 176), a = 13.1028(5) Å, c = 15.2028(6) Å, V = 2260.38(19) Å^3, Z = 18, T = 293(2) K, μ(synchrotron) = 0.224 mm^{-1}, $Dcalc$ = 1.613 g/cm^3, 3045 reflections measured (4.334° ≤ 2Θ ≤ 31.934°), 1063 unique (R_{int} = 0.0348, R_{sigma} = 0.0448) which were used in all calculations. The final R_1 was 0.0335 (I > 2σ(I)) and wR_2 was 0.0987 (all data).

Refinement model description

Number of restraints - 0, number of constraints - unknown.
Details:
N/A

This report has been created with Olex2, compiled on 2023.03.06 svn.rbb2c1857 for OlexSys. Please let us know if there are any errors or if you would like to have additional features.

$AlPO_4$-17/O_2 1.66GPa

Table 1 Crystal data and structure refinement for 17O2_sc2_1p66GPa.

Identification code	17O2_sc2_1p66GPa
Empirical formula	AlO_4P
Formula weight	121.95
Temperature/K	293(2)
Crystal system	hexagonal
Space group	$P6_3/m$
a/Å	13.0949(9)
b/Å	13.0949(9)
c/Å	15.1539(10)
α/°	90
β/°	90
γ/°	120
Volume/Å^3	2250.4(3)
Z	18
ρ_{calc}g/cm^3	1.620
μ/mm^{-1}	0.225
F(000)	1080.0
Crystal size/mm^3	0.125 × 0.050 × 0.050
Radiation	synchrotron (λ = 0.49555)
2Θ range for data collection/°	4.338 to 31.96
Index ranges	$-11 \leq h \leq 11$, $-11 \leq k \leq 11$, $-16 \leq l \leq 16$
Reflections collected	3327
Independent reflections	1067 [R_{int} = 0.0289, R_{sigma} = 0.0324]
Data/restraints/parameters	1067/0/88
Goodness-of-fit on F^2	1.092
Final R indexes [I>=2σ (I)]	R_1 = 0.0426, wR_2 = 0.1220
Final R indexes [all data]	R_1 = 0.0514, wR_2 = 0.1261
Largest diff. peak/hole / e Å^{-3}	0.28/-0.33

Table 2 Fractional Atomic Coordinates (×10^4) and Equivalent Isotropic Displacement Parameters (Å^2×10^3) for 17O2_sc2_1p66GPa. U_{eq} is defined as 1/3 of the trace of the orthogonalised U_{IJ} tensor.

Atom	*x*	*y*	*z*	U(eq)
P1	6663.9(13)	10918.3(14)	7500	28.3(4)
P2	7617.8(10)	7639.6(10)	6031.6(7)	29.8(4)
Al2	6692.2(15)	5766.2(15)	7500	27.4(5)
Al1	7643.2(11)	10023.2(11)	6022.8(8)	29.3(4)
O1	5490(3)	10848(4)	7500	35.4(10)

Table 2 Fractional Atomic Coordinates (×10^4) and Equivalent Isotropic Displacement Parameters (Å^2×10^3) for 17O2_sc2_1p66GPa. U_{eq} is defined as 1/3 of the trace of the orthogonalised U_{IJ} tensor.

Atom	*x*	*y*	*z*	U(eq)
O4	7487(3)	8683(3)	6293(2)	39.1(8)
O5	6809(3)	6589(3)	6583.7(19)	41.3(8)
O2	7629(4)	12197(4)	7500	44.2(12)
O6	7270(3)	7308(3)	5069.4(18)	45.2(9)
O3	6762(3)	10309(3)	6686.7(19)	42.5(8)
O7	8892(3)	7968(3)	6182(2)	47.1(9)

Table 3 Anisotropic Displacement Parameters (Å^2×10^3) for 17O2_sc2_1p66GPa. The Anisotropic displacement factor exponent takes the form: -2π^2[h^{2}a*^{2}U$_{11}$+2hka*b*U$_{12}$+…].

Atom	U_{11}	U_{22}	U_{33}	U_{23}	U_{13}	U_{12}
P1	25.2(9)	29.1(9)	31.9(9)	0	0	14.7(7)
P2	32.2(7)	29.8(7)	29.6(7)	2.8(5)	1.9(5)	17.2(6)
Al2	25.0(10)	24.0(10)	30.1(10)	0	0	10.0(8)
Al1	32.7(8)	28.0(8)	27.5(8)	-0.2(5)	0.8(5)	15.4(6)
O1	29(2)	44(3)	40(2)	0	0	23(2)
O4	47(2)	31.8(18)	43.7(18)	3.5(14)	6.8(14)	23.9(16)
O5	50(2)	37.0(19)	34.9(17)	8.2(13)	5.1(14)	19.7(16)
O2	28(3)	29(3)	68(3)	0	0	8(2)
O6	54(2)	56(2)	22.5(17)	-1.3(14)	-0.3(14)	25.7(18)
O3	48(2)	48(2)	37.6(18)	-3.0(15)	4.4(15)	28.6(17)
O7	37.9(19)	50(2)	58(2)	-10.8(16)	-3.9(15)	25.9(17)

Table 4 Bond Lengths for 17O2_sc2_1p66GPa.

Atom	Atom	Length/Å	Atom	Atom	Length/Å
P1	O1	1.494(4)	Al2	O5^1	1.717(3)
P1	O2	1.512(4)	Al2	O2^3	1.716(4)
P1	O3^1	1.508(3)	Al1	O4	1.712(3)
P1	O3	1.508(3)	Al1	O6^4	1.715(3)
P2	O4	1.512(3)	Al1	O3	1.708(3)
P2	O5	1.502(3)	Al1	O7^5	1.712(3)
P2	O6	1.525(3)	O1	Al2^6	1.698(4)
P2	O7	1.517(3)	O2	Al2^5	1.716(4)
Al2	O1^2	1.698(4)	O6	Al1^7	1.715(3)
Al2	O5	1.717(3)	O7	Al1^3	1.712(3)

^{1}+X,+Y,3/2-Z; ^{2}+Y-X,1-X,+Z; ^{3}2-Y,1+X-Y,+Z; ^{4}+Y,1-X+Y,1-Z; ^{5}1+Y-X,2-X,+Z; ^{6}1-Y,1+X-Y,+Z; ^{7}1-Y+X,+X,1-Z

Table 5 Bond Angles for 17O2_sc2_1p66GPa.

Atom	Atom	Atom	Angle/°	Atom	Atom	Atom	Angle/°
O1	P1	O2	109.4(3)	O2[3]	Al2	O5[1]	109.83(15)
O1	P1	O3	108.94(16)	O2[3]	Al2	O5	109.83(15)
O1	P1	O3[1]	108.94(16)	O4	Al1	O6[4]	110.02(16)
O3	P1	O2	109.94(17)	O4	Al1	O7[5]	109.69(16)
O3[1]	P1	O2	109.94(17)	O3	Al1	O4	109.67(16)
O3	P1	O3[1]	109.6(3)	O3	Al1	O6[4]	111.17(17)
O4	P2	O6	110.75(18)	O3	Al1	O7[5]	108.40(17)
O4	P2	O7	108.07(19)	O7[5]	Al1	O6[4]	107.85(18)
O5	P2	O4	109.59(18)	P1	O1	Al2[6]	174.0(3)
O5	P2	O6	107.37(19)	P2	O4	Al1	148.7(2)
O5	P2	O7	110.36(19)	P2	O5	Al2	145.7(2)
O7	P2	O6	110.72(19)	P1	O2	Al2[5]	153.6(3)
O1[2]	Al2	O5[1]	109.49(14)	P2	O6	Al1[7]	147.8(3)
O1[2]	Al2	O5	109.49(14)	P1	O3	Al1	147.4(2)
O1[2]	Al2	O2[3]	110.2(2)	P2	O7	Al1[3]	147.6(2)
O5[1]	Al2	O5	107.9(2)				

[1]+X,+Y,3/2-Z; [2]+Y-X,1-X,+Z; [3]2-Y,1+X-Y,+Z; [4]+Y,1-X+Y,1-Z; [5]1+Y-X,2-X,+Z; [6]1-Y,1+X-Y,+Z; [7]1-Y+X,+X,1-Z

Table 6 Torsion Angles for 17O2_sc2_1p66GPa.

A	B	C	D	Angle/°	A	B	C	D	Angle/°
O1	P1	O2	Al2[1]	180.000(0)	O6	P2	O5	Al2	142.7(4)
O1	P1	O3	Al1	169.7(4)	O6	P2	O7	Al1[4]	-47.4(5)
O1[2]	Al2	O5	P2	-170.4(4)	O6[6]	Al1	O4	P2	37.1(5)
O4	P2	O5	Al2	-96.9(4)	O6[6]	Al1	O3	P1	-140.7(4)
O4	P2	O6	Al1[3]	73.4(4)	O3	P1	O2	Al2[1]	-60.39(15)
O4	P2	O7	Al1[4]	-168.8(4)	O3[5]	P1	O2	Al2[1]	60.39(15)
O4	Al1	O3	P1	97.4(4)	O3[5]	P1	O3	Al1	-71.2(5)
O5	P2	O4	Al1	-157.9(4)	O3	Al1	O4	P2	159.7(4)
O5	P2	O6	Al1[3]	-167.0(4)	O7	P2	O4	Al1	81.8(5)
O5	P2	O7	Al1[4]	71.4(5)	O7	P2	O5	Al2	22.0(4)
O5[5]	Al2	O5	P2	70.4(5)	O7	P2	O6	Al1[3]	-46.4(5)
O2	P1	O3	Al1	49.7(5)	O7[1]	Al1	O4	P2	-81.3(5)
O2[4]	Al2	O5	P2	-49.3(4)	O7[1]	Al1	O3	P1	-22.3(5)
O6	P2	O4	Al1	-39.7(5)					

[1]1+Y-X,2-X,+Z; [2]+Y-X,1-X,+Z; [3]1-Y+X,+X,1-Z; [4]2-Y,1+X-Y,+Z; [5]+X,+Y,3/2-Z; [6]+Y,1-X+Y,1-Z

Table 7 Solvent masks information for 17O2_sc2_1p66GPa.

Number	X	Y	Z	Volume	Electron count	Content
1	0.000	0.000	0.000	11.0	5.4	?
2	0.000	0.000	0.250	20.4	25.8	?
3	0.000	0.000	0.500	11.0	5.4	?
4	0.000	0.000	0.750	20.4	25.8	?
5	-0.979	-0.127	-0.729	893.3	302.2	?

Experimental

Single crystals of AlO_4P **[17O2_sc2_1p66GPa]** were **[]**. A suitable crystal was selected and **[]** on a **esperanto-CrysAlisPro-abstract goniometer imported esperanto images** diffractometer. The crystal was kept at 293(2) K during data collection. Using Olex2 [1], the structure was solved with the SHELXT [2] structure solution program using Intrinsic Phasing and refined with the SHELXL [3] refinement package using Least Squares minimisation.

1. Dolomanov, O.V., Bourhis, L.J., Gildea, R.J, Howard, J.A.K. & Puschmann, H. (2009), J. Appl. Cryst. 42, 339-341.
2. Sheldrick, G.M. (2015). Acta Cryst. A71, 3-8.
3. Sheldrick, G.M. (2015). Acta Cryst. C71, 3-8.

Crystal structure determination of [17O2_sc2_1p66GPa]

Crystal Data for AlO_4P (M =121.95 g/mol): hexagonal, space group $P6_3/m$ (no. 176), a = 13.0949(9) Å, c = 15.1539(10) Å, V = 2250.4(3) Å^3, Z = 18, T = 293(2) K, μ(synchrotron) = 0.225 mm^{-1}, $Dcalc$ = 1.620 g/cm^3, 3327 reflections measured (4.338° ≤ 2Θ ≤ 31.96°), 1067 unique (R_{int} = 0.0289, R_{sigma} = 0.0324) which were used in all calculations. The final R_1 was 0.0426 (I > 2σ(I)) and wR_2 was 0.1261 (all data).

Refinement model description

Number of restraints - 0, number of constraints - unknown.
Details:
N/A

This report has been created with Olex2, compiled on 2023.03.06 svn.rbb2c1857 for OlexSys. Please let us know if there are any errors or if you would like to have additional features.

$AlPO_4$-17/O_2 2.3GPa

Table 1 Crystal data and structure refinement for 17O2_sc2_2p3GPa.

Identification code	17O2_sc2_2p3GPa
Empirical formula	AlO_4P
Formula weight	121.95
Temperature/K	293(2)
Crystal system	hexagonal
Space group	$P6_3/m$
a/Å	13.0992(6)
b/Å	13.0992(6)
c/Å	15.1105(7)
α/°	90
β/°	90
γ/°	120
Volume/Å^3	2245.4(2)
Z	18
ρ_{calc}g/cm^3	1.623
μ/mm^{-1}	0.225
F(000)	1080.0
Crystal size/mm^3	0.125 × 0.050 × 0.050
Radiation	synchrotron (λ = 0.49555)
2Θ range for data collection/°	4.336 to 31.918
Index ranges	$-11 \le h \le 11$, $-11 \le k \le 10$, $-16 \le l \le 16$
Reflections collected	3019
Independent reflections	1053 [R_{int} = 0.0262, R_{sigma} = 0.0345]
Data/restraints/parameters	1053/0/88
Goodness-of-fit on F^2	1.095
Final R indexes [I>=2σ (I)]	R_1 = 0.0340, wR_2 = 0.0977
Final R indexes [all data]	R_1 = 0.0415, wR_2 = 0.1002
Largest diff. peak/hole / e Å^{-3}	0.31/-0.27

Table 2 Fractional Atomic Coordinates (×10^4) and Equivalent Isotropic Displacement Parameters (Å^2×10^3) for 17O2_sc2_2p3GPa. U_{eq} is defined as 1/3 of the trace of the orthogonalised U_{IJ} tensor.

Atom	*x*	*y*	*z*	U(eq)
P1	6664.0(10)	10914.8(10)	7500	23.2(4)
P2	7613.8(7)	7634.6(7)	6039.0(5)	24.0(3)
Al2	6686.1(11)	5766.8(11)	7500	22.2(4)
Al1	7640.9(8)	10021.2(8)	6028.0(6)	23.7(3)
O1	5498(3)	10873(3)	7500	28.7(8)

Table 2 Fractional Atomic Coordinates (×10^4) and Equivalent Isotropic Displacement Parameters (Å^2×10^3) for 17O2_sc2_2p3GPa. U_{eq} is defined as 1/3 of the trace of the orthogonalised U_{IJ} tensor.

Atom	*x*	*y*	*z*	U(eq)
O4	7493(2)	8685.8(19)	6306.1(14)	33.1(6)
O5	6784(2)	6583.2(19)	6578.9(14)	35.6(6)
O2	7649(3)	12174(3)	7500	42.9(9)
O6	7303(2)	7332(2)	5071.1(13)	37.2(6)
O3	6737(2)	10279(2)	6683.4(13)	37.6(6)
O7	8877(2)	7938(2)	6209.3(16)	43.9(7)

Table 3 Anisotropic Displacement Parameters (Å^2×10^3) for 17O2_sc2_2p3GPa. The Anisotropic displacement factor exponent takes the form: -2π^2[h^{2}a*^{2}U_{11}+2hka*b*U_{12}+…].

Atom	U_{11}	U_{22}	U_{33}	U_{23}	U_{13}	U_{12}
P1	19.5(7)	24.2(7)	27.3(6)	0	0	12.0(6)
P2	25.5(5)	24.0(5)	24.1(5)	2.4(3)	1.4(3)	13.7(4)
Al2	20.0(8)	19.2(8)	24.7(7)	0	0	7.7(6)
Al1	25.6(6)	21.0(6)	24.9(6)	-0.8(4)	2.3(4)	11.8(5)
O1	24.2(18)	31.0(19)	32.6(17)	0	0	15.2(16)
O4	41.5(15)	25.6(14)	37.9(12)	4.1(10)	4.7(11)	21.2(13)
O5	45.4(16)	27.4(14)	29.9(12)	7.7(10)	6.5(10)	15.2(12)
O2	29(2)	28(2)	66(2)	0	0	10.6(18)
O6	40.9(15)	41.8(15)	25.7(12)	0.9(10)	-0.7(10)	18.4(13)
O3	42.3(16)	44.5(15)	33.4(13)	-5.1(11)	5.5(11)	27.3(13)
O7	33.9(16)	48.9(17)	55.6(16)	-11.7(12)	-6.2(12)	25.7(14)

Table 4 Bond Lengths for 17O2_sc2_2p3GPa.

Atom	Atom	Length/Å	Atom	Atom	Length/Å
P1	O1	1.501(3)	Al2	O5^1	1.720(2)
P1	O2	1.503(3)	Al2	O2^3	1.716(3)
P1	O3^1	1.518(2)	Al1	O4	1.713(2)
P1	O3	1.518(2)	Al1	O6^4	1.711(2)
P2	O4	1.517(2)	Al1	O3	1.702(2)
P2	O5	1.499(2)	Al1	O7^5	1.713(2)
P2	O6	1.517(2)	O1	Al2^6	1.688(3)
P2	O7	1.518(2)	O2	Al2^5	1.717(3)
Al2	O1^2	1.688(3)	O6	Al1^7	1.711(2)
Al2	O5	1.720(2)	O7	Al1^3	1.713(2)

^{1}+X,+Y,3/2-Z; ^{2}+Y-X,1-X,+Z; ^{3}2-Y,1+X-Y,+Z; ^{4}+Y,1-X+Y,1-Z; ^{5}1+Y-X,2-X,+Z; ^{6}1-Y,1+X-Y,+Z; ^{7}1-Y+X,+X,1-Z

Table 5 Bond Angles for 17O2_sc2_2p3GPa.

Atom	Atom	Atom	Angle/°	Atom	Atom	Atom	Angle/°
O1	P1	O2	109.82(19)	O2^{3}	Al2	O5^{1}	109.62(11)
O1	P1	O3^{1}	108.79(12)	O2^{3}	Al2	O5	109.62(11)
O1	P1	O3	108.78(12)	O6^{4}	Al1	O4	109.77(12)
O2	P1	O3	110.33(13)	O6^{4}	Al1	O7^{5}	108.09(12)
O2	P1	O3^{1}	110.33(13)	O3	Al1	O4	108.99(12)
O3	P1	O3^{1}	108.74(19)	O3	Al1	O6^{4}	111.93(13)
O4	P2	O7	108.16(14)	O3	Al1	O7^{5}	108.05(13)
O5	P2	O4	109.42(13)	O7^{5}	Al1	O4	109.99(12)
O5	P2	O6	107.95(14)	P1	O1	Al2^{6}	176.4(2)
O5	P2	O7	110.05(14)	P2	O4	Al1	148.39(15)
O6	P2	O4	110.95(12)	P2	O5	Al2	143.70(16)
O6	P2	O7	110.31(14)	P1	O2	Al2^{5}	156.9(2)
O1^{2}	Al2	O5^{1}	109.47(10)	P2	O6	Al1^{7}	150.77(18)
O1^{2}	Al2	O5	109.47(10)	P1	O3	Al1	145.34(16)
O1^{2}	Al2	O2^{3}	110.63(17)	P2	O7	Al1^{3}	148.23(17)
O5^{1}	Al2	O5	107.99(17)				

^{1}+X,+Y,3/2-Z; ^{2}+Y-X,1-X,+Z; ^{3}2-Y,1+X-Y,+Z; ^{4}+Y,1-X+Y,1-Z; ^{5}1+Y-X,2-X,+Z; ^{6}1-Y,1+X-Y,+Z; ^{7}1-Y+X,+X,1-Z

Table 6 Torsion Angles for 17O2_sc2_2p3GPa.

A	B	C	D	Angle/°	A	B	C	D	Angle/°
O1	P1	O2	Al2^{1}	180.000(0)	O6	P2	O5	Al2	141.8(3)
O1	P1	O3	Al1	166.8(3)	O6	P2	O7	Al1^{4}	-42.5(4)
O1^{2}	Al2	O5	P2	-168.9(3)	O6^{6}	Al1	O4	P2	34.1(3)
O4	P2	O5	Al2	-97.4(3)	O6^{6}	Al1	O3	P1	-139.1(3)
O4	P2	O6	Al1^{3}	71.4(3)	O3^{5}	P1	O2	Al2^{1}	60.09(11)
O4	P2	O7	Al1^{4}	-164.0(3)	O3	P1	O2	Al2^{1}	-60.09(11)
O4	Al1	O3	P1	99.3(3)	O3^{5}	P1	O3	Al1	-74.8(3)
O5	P2	O4	Al1	-155.0(3)	O3	Al1	O4	P2	157.0(3)
O5	P2	O6	Al1^{3}	-168.7(3)	O7	P2	O4	Al1	85.1(3)
O5	P2	O7	Al1^{4}	76.5(4)	O7	P2	O5	Al2	21.3(3)
O5^{5}	Al2	O5	P2	72.0(3)	O7	P2	O6	Al1^{3}	-48.4(3)
O2	P1	O3	Al1	46.3(3)	O7^{1}	Al1	O4	P2	-84.7(3)
O2^{4}	Al2	O5	P2	-47.4(3)	O7^{1}	Al1	O3	P1	-20.2(3)
O6	P2	O4	Al1	-36.0(3)					

^{1}1+Y-X,2-X,+Z; ^{2}+Y-X,1-X,+Z; ^{3}1-Y+X,+X,1-Z; ^{4}2-Y,1+X-Y,+Z; ^{5}+X,+Y,3/2-Z; ^{6}+Y,1-X+Y,1-Z

Table 7 Solvent masks information for 17O2_sc2_2p3GPa.

Number	X	Y	Z	Volume	Electron count	Content
1	0.000	0.000	0.000	12.8	10.2	?
2	0.000	0.000	0.250	19.8	26.4	?
3	0.000	0.000	0.500	12.8	10.2	?
4	0.000	0.000	0.750	19.8	26.4	?
5	-0.922	-0.349	0.131	898.8	341.3	?

Experimental

Single crystals of AlO_4P **[17O2_sc2_2p3GPa]** were **[]**. A suitable crystal was selected and **[]** on a **esperanto-CrysAlisPro-abstract goniometer imported esperanto images** diffractometer. The crystal was kept at 293(2) K during data collection. Using Olex2 [1], the structure was solved with the SHELXT [2] structure solution program using Intrinsic Phasing and refined with the SHELXL [3] refinement package using Least Squares minimisation.

1. Dolomanov, O.V., Bourhis, L.J., Gildea, R.J, Howard, J.A.K. & Puschmann, H. (2009), J. Appl. Cryst. 42, 339-341.
2. Sheldrick, G.M. (2015). Acta Cryst. A71, 3-8.
3. Sheldrick, G.M. (2015). Acta Cryst. C71, 3-8.

Crystal structure determination of [17O2_sc2_2p3GPa]

Crystal Data for AlO_4P (M =121.95 g/mol): hexagonal, space group $P6_3/m$ (no. 176), a = 13.0992(6) Å, c = 15.1105(7) Å, V = 2245.4(2) Å^3, Z = 18, T = 293(2) K, μ(synchrotron) = 0.225 mm^{-1}, $Dcalc$ = 1.623 g/cm^3, 3019 reflections measured (4.336° ≤ 2Θ ≤ 31.918°), 1053 unique (R_{int} = 0.0262, R_{sigma} = 0.0345) which were used in all calculations. The final R_1 was 0.0340 (I > 2σ(I)) and wR_2 was 0.1002 (all data).

Refinement model description

Number of restraints - 0, number of constraints - unknown.
Details:
N/A

This report has been created with Olex2, compiled on 2023.03.06 svn.rbb2c1857 for OlexSys. Please let us know if there are any errors or if you would like to have additional features.

$AlPO_4$-17/O_2 3.14GPa

Table 1 Crystal data and structure refinement for 17O2_sc2_3p14GPa.

Identification code	17O2_sc2_3p14GPa
Empirical formula	AlO_4P
Formula weight	121.95
Temperature/K	293(2)
Crystal system	hexagonal
Space group	$P6_3/m$
a/Å	13.0853(7)
b/Å	13.0853(7)
c/Å	15.0355(8)
α/°	90
β/°	90
γ/°	120
Volume/$Å^3$	2229.5(3)
Z	18
$\rho_{calc}g/cm^3$	1.635
μ/mm^{-1}	0.227
F(000)	1080.0
Crystal size/mm^3	0.125 × 0.050 × 0.050
Radiation	synchrotron (λ = 0.49555)
2Θ range for data collection/°	4.34 to 31.948
Index ranges	$-11 \le h \le 11$, $-11 \le k \le 10$, $-16 \le l \le 16$
Reflections collected	3017
Independent reflections	1057 [R_{int} = 0.0254, R_{sigma} = 0.0338]
Data/restraints/parameters	1057/0/88
Goodness-of-fit on F^2	1.044
Final R indexes [I>=2σ (I)]	R_1 = 0.0358, wR_2 = 0.0997
Final R indexes [all data]	R_1 = 0.0443, wR_2 = 0.1031
Largest diff. peak/hole / e $Å^{-3}$	0.28/-0.27

Table 2 Fractional Atomic Coordinates (×10^4) and Equivalent Isotropic Displacement Parameters ($Å^2$×10^3) for 17O2_sc2_3p14GPa. U_{eq} is defined as 1/3 of the trace of the orthogonalised U_{IJ} tensor.

Atom	*x*	*y*	*z*	U(eq)
P1	6665.9(10)	10913.3(11)	7500	23.5(4)
P2	7612.2(8)	7636.2(8)	6046.6(5)	24.7(3)
Al2	6677.5(11)	5766.9(12)	7500	22.9(4)
Al1	7641.8(9)	10022.0(8)	6032.8(6)	24.3(3)
O1	5510(3)	10904(3)	7500	30.8(8)

Table 2 Fractional Atomic Coordinates (×10^4) and Equivalent Isotropic Displacement Parameters (Å^2×10^3) for 17O2_sc2_3p14GPa. U_{eq} is defined as 1/3 of the trace of the orthogonalised U_{IJ} tensor.

Atom	*x*	*y*	*z*	U(eq)
O4	7510(2)	8695(2)	6319.2(14)	34.8(6)
O5	6759(2)	6577(2)	6578.0(15)	38.5(7)
O2	7666(3)	12165(3)	7500	45.2(10)
O6	7320(2)	7353(2)	5070.3(13)	38.1(7)
O3	6718(2)	10267(2)	6680.3(14)	39.0(7)
O7	8861(2)	7915(3)	6239.1(16)	44.9(7)

Table 3 Anisotropic Displacement Parameters (Å^2×10^3) for 17O2_sc2_3p14GPa. The Anisotropic displacement factor exponent takes the form: -2π^2[h^{2}a*^{2}U$_{11}$+2hka*b*U$_{12}$+…].

Atom	U_{11}	U_{22}	U_{33}	U_{23}	U_{13}	U_{12}
P1	19.9(7)	24.7(7)	28.4(7)	0	0	13.1(6)
P2	27.7(6)	25.7(6)	23.5(5)	2.1(4)	1.1(4)	15.5(4)
Al2	20.8(9)	20.0(8)	25.1(7)	0	0	8.2(7)
Al1	29.1(6)	22.2(6)	22.3(6)	-1.0(4)	1.9(4)	13.4(5)
O1	25(2)	35(2)	35.5(18)	0	0	17.5(17)
O4	44.6(16)	28.4(14)	37.3(13)	5.7(10)	6.6(11)	22.7(13)
O5	48.8(17)	31.4(15)	30.8(12)	8.7(10)	7.4(11)	16.7(13)
O2	30(2)	28(2)	72(3)	0	0	10.2(18)
O6	47.2(16)	47.1(17)	19.7(13)	0.5(10)	-1.1(10)	23.3(14)
O3	42.8(17)	48.7(16)	33.7(14)	-3.4(12)	5.7(11)	29.0(14)
O7	35.2(16)	53.7(18)	52.9(16)	-10.5(13)	-4.5(12)	27.6(15)

Table 4 Bond Lengths for 17O2_sc2_3p14GPa.

Atom	Atom	Length/Å	Atom	Atom	Length/Å
P1	O1	1.506(3)	Al2	O5^1	1.716(2)
P1	O2	1.500(4)	Al2	O2^3	1.715(4)
P1	O3^1	1.515(2)	Al1	O4	1.712(2)
P1	O3	1.515(2)	Al1	O6^4	1.703(2)
P2	O4	1.513(2)	Al1	O3	1.703(2)
P2	O5	1.503(2)	Al1	O7^5	1.717(3)
P2	O6	1.515(2)	O1	Al2^6	1.676(3)
P2	O7	1.513(3)	O2	Al2^5	1.715(4)
Al2	O1^2	1.676(3)	O6	Al1^7	1.703(2)
Al2	O5	1.716(2)	O7	Al1^3	1.717(3)

^{1}+X,+Y,3/2-Z; ^{2}+Y-X,1-X,+Z; ^{3}2-Y,1+X-Y,+Z; ^{4}+Y,1-X+Y,1-Z; ^{5}1+Y-X,2-X,+Z; ^{6}1-Y,1+X-Y,+Z; ^{7}1-Y+X,+X,1-Z

Table 5 Bond Angles for 17O2_sc2_3p14GPa.

Atom	Atom	Atom	Angle/°	Atom	Atom	Atom	Angle/°
O1	P1	O3	108.69(12)	O2[3]	Al2	O5[1]	109.69(12)
O1	P1	O3[1]	108.69(12)	O2[3]	Al2	O5	109.69(12)
O2	P1	O1	109.5(2)	O4	Al1	O7[4]	109.72(14)
O2	P1	O3[1]	110.55(13)	O6[5]	Al1	O4	110.11(12)
O2	P1	O3	110.55(13)	O6[5]	Al1	O3	111.95(13)
O3	P1	O3[1]	108.8(2)	O6[5]	Al1	O7[4]	108.51(13)
O4	P2	O6	111.03(13)	O3	Al1	O4	109.04(13)
O4	P2	O7	107.82(15)	O3	Al1	O7[4]	107.46(13)
O5	P2	O4	109.58(14)	P1	O1	Al2[6]	179.4(2)
O5	P2	O6	108.02(15)	P2	O4	Al1	148.23(16)
O5	P2	O7	109.61(15)	P2	O5	Al2	141.92(18)
O7	P2	O6	110.77(14)	P1	O2	Al2[4]	159.0(3)
O1[2]	Al2	O5[1]	109.74(11)	P2	O6	Al1[7]	152.54(19)
O1[2]	Al2	O5	109.74(11)	P1	O3	Al1	143.52(17)
O1[2]	Al2	O2[3]	110.16(18)	P2	O7	Al1[3]	148.12(18)
O5[1]	Al2	O5	107.79(18)				

[1]+X,+Y,3/2-Z; [2]+Y-X,1-X,+Z; [3]2-Y,1+X-Y,+Z; [4]1+Y-X,2-X,+Z; [5]+Y,1-X+Y,1-Z; [6]1-Y,1+X-Y,+Z; [7]1-Y+X,+X,1-Z

Table 6 Torsion Angles for 17O2_sc2_3p14GPa.

A	B	C	D	Angle/°	A	B	C	D	Angle/°
O1	P1	O2	Al2[1]	180.000(0)	O6	P2	O5	Al2	141.4(3)
O1	P1	O3	Al1	165.5(3)	O6	P2	O7	Al1[4]	-37.5(4)
O1[2]	Al2	O5	P2	-167.2(3)	O6[6]	Al1	O4	P2	30.4(4)
O4	P2	O5	Al2	-97.5(3)	O6[6]	Al1	O3	P1	-138.8(3)
O4	P2	O6	Al1[3]	70.8(4)	O3	P1	O2	Al2[1]	-60.30(12)
O4	P2	O7	Al1[4]	-159.2(3)	O3[5]	P1	O2	Al2[1]	60.30(12)
O4	Al1	O3	P1	99.1(3)	O3[5]	P1	O3	Al1	-76.2(4)
O5	P2	O4	Al1	-151.4(3)	O3	Al1	O4	P2	153.6(3)
O5	P2	O6	Al1[3]	-169.0(3)	O7	P2	O4	Al1	89.4(3)
O5	P2	O7	Al1[4]	81.6(4)	O7	P2	O5	Al2	20.6(3)
O5[5]	Al2	O5	P2	73.3(3)	O7	P2	O6	Al1[3]	-48.9(4)
O2	P1	O3	Al1	45.4(3)	O7[1]	Al1	O4	P2	-89.0(3)
O2[4]	Al2	O5	P2	-46.1(3)	O7[1]	Al1	O3	P1	-19.7(3)
O6	P2	O4	Al1	-32.2(4)					

[1]1+Y-X,2-X,+Z; [2]+Y-X,1-X,+Z; [3]1-Y+X,+X,1-Z; [4]2-Y,1+X-Y,+Z; [5]+X,+Y,3/2-Z; [6]+Y,1-X+Y,1-Z

Table 7 Solvent masks information for 17O2_sc2_3p14GPa.

Number	X	Y	Z	Volume	Electron count	Content
1	0.000	0.000	0.000	13.4	15.5	?
2	0.000	0.000	0.250	19.6	26.9	?
3	0.000	0.000	0.500	13.4	15.5	?
4	0.000	0.000	0.750	19.6	26.9	?
5	-0.787	-0.842	0.102	887.3	354.7	?

Experimental

Single crystals of AlO_4P **[17O2_sc2_3p14GPa]** were **[]**. A suitable crystal was selected and **[]** on a **esperanto-CrysAlisPro-abstract goniometer imported esperanto images** diffractometer. The crystal was kept at 293(2) K during data collection. Using Olex2 [1], the structure was solved with the SHELXT [2] structure solution program using Intrinsic Phasing and refined with the SHELXL [3] refinement package using Least Squares minimisation.

1. Dolomanov, O.V., Bourhis, L.J., Gildea, R.J, Howard, J.A.K. & Puschmann, H. (2009), J. Appl. Cryst. 42, 339-341.
2. Sheldrick, G.M. (2015). Acta Cryst. A71, 3-8.
3. Sheldrick, G.M. (2015). Acta Cryst. C71, 3-8.

Crystal structure determination of [17O2_sc2_3p14GPa]

Crystal Data for AlO_4P (M =121.95 g/mol): hexagonal, space group $P6_3/m$ (no. 176), a = 13.0853(7) Å, c = 15.0355(8) Å, V = 2229.5(3) Å^3, Z = 18, T = 293(2) K, μ(synchrotron) = 0.227 mm^{-1}, $Dcalc$ = 1.635 g/cm^3, 3017 reflections measured (4.34° ≤ 2Θ ≤ 31.948°), 1057 unique (R_{int} = 0.0254, R_{sigma} = 0.0338) which were used in all calculations. The final R_1 was 0.0358 (I > 2σ(I)) and wR_2 was 0.1031 (all data).

Refinement model description

Number of restraints - 0, number of constraints - unknown.
Details:
N/A

This report has been created with Olex2, compiled on 2023.03.06 svn.rbb2c1857 for OlexSys. Please let us know if there are any errors or if you would like to have additional features.

$AlPO_4$-17/O_2 3.459GPa

Table 1 Crystal data and structure refinement for 17O2_sc2_3p459GPa.

Identification code	17O2_sc2_3p459GPa
Empirical formula	AlO_4P
Formula weight	121.95
Temperature/K	293(2)
Crystal system	hexagonal
Space group	$P6_3/m$
a/Å	13.0957(6)
b/Å	13.0957(6)
c/Å	15.0060(7)
α/°	90
β/°	90
γ/°	120
Volume/Å^3	2228.7(2)
Z	18
ρ_{calc}g/cm^3	1.636
μ/mm^{-1}	0.227
F(000)	1080.0
Crystal size/mm^3	0.125 × 0.050 × 0.050
Radiation	synchrotron (λ = 0.49555)
2Θ range for data collection/°	4.338 to 31.908
Index ranges	$-11 \leq h \leq 11$, $-11 \leq k \leq 10$, $-16 \leq l \leq 16$
Reflections collected	2963
Independent reflections	1050 [R_{int} = 0.0252, R_{sigma} = 0.0344]
Data/restraints/parameters	1050/0/88
Goodness-of-fit on F^2	1.099
Final R indexes [I>=2σ (I)]	R_1 = 0.0367, wR_2 = 0.1107
Final R indexes [all data]	R_1 = 0.0466, wR_2 = 0.1155
Largest diff. peak/hole / e Å^{-3}	0.27/-0.30

Table 2 Fractional Atomic Coordinates (×10^4) and Equivalent Isotropic Displacement Parameters (Å^2×10^3) for 17O2_sc2_3p459GPa. U_{eq} is defined as 1/3 of the trace of the orthogonalised U_{IJ} tensor.

Atom	*x*	*y*	*z*	U(eq)
P1	6665.7(10)	10908.7(11)	7500	21.3(4)
P2	7609.9(8)	7633.9(8)	6053.1(5)	22.8(3)
Al2	6675.3(12)	5771.2(12)	7500	21.4(4)
Al1	7639.4(9)	10020.1(9)	6038.4(6)	22.4(4)
O1	5520(3)	10918(3)	7500	29.1(9)

Table 2 Fractional Atomic Coordinates (×10[4]) and Equivalent Isotropic Displacement Parameters (Å[2]×10[3]) for 17O2_sc2_3p459GPa. U_{eq} is defined as 1/3 of the trace of the orthogonalised U_{IJ} tensor.

Atom	*x*	*y*	*z*	U(eq)
O4	7518(2)	8698(2)	6325.6(15)	32.1(7)
O5	6734(2)	6571(2)	6571.2(15)	33.7(7)
O2	7683(3)	12150(3)	7500	38.2(10)
O6	7334(2)	7367(2)	5071.1(13)	36.1(7)
O3	6699(2)	10255(2)	6672.8(14)	35.8(7)
O7	8851(2)	7890(2)	6257.4(17)	40.5(8)

Table 3 Anisotropic Displacement Parameters (Å[2]×10[3]) for 17O2_sc2_3p459GPa. The Anisotropic displacement factor exponent takes the form: -2π[2][h[2]a*[2]U11+2hka*b*U12+…].

Atom	U_{11}	U_{22}	U_{33}	U_{23}	U_{13}	U_{12}
P1	18.3(8)	23.4(8)	23.6(7)	0	0	11.4(6)
P2	27.2(6)	24.1(6)	19.6(6)	2.2(4)	1.0(4)	14.6(5)
Al2	21.0(9)	19.0(9)	21.1(7)	0	0	7.7(7)
Al1	26.9(7)	21.7(7)	18.8(6)	-1.5(4)	1.5(4)	12.3(5)
O1	25(2)	31(2)	32.8(18)	0	0	15.1(17)
O4	42.8(17)	24.8(15)	34.0(13)	3.1(11)	4.8(11)	20.8(14)
O5	40.3(17)	27.1(15)	26.9(12)	8.9(10)	6.3(10)	11.7(13)
O2	21(2)	26(2)	63(3)	0	0	9.3(18)
O6	44.2(17)	45.4(18)	18.3(13)	0.8(10)	-1.2(10)	22.1(15)
O3	35.9(17)	42.9(17)	32.0(14)	-7.0(12)	4.3(11)	22.1(14)
O7	31.6(17)	48.3(19)	47.2(16)	-7.1(13)	-1.8(12)	24.0(15)

Table 4 Bond Lengths for 17O2_sc2_3p459GPa.

Atom	Atom	Length/Å	Atom	Atom	Length/Å
P1	O1	1.507(3)	Al2	O5	1.722(2)
P1	O2	1.500(3)	Al2	O2[3]	1.716(4)
P1	O3	1.521(2)	Al1	O4	1.712(2)
P1	O3[1]	1.521(2)	Al1	O6[4]	1.705(2)
P2	O4	1.514(2)	Al1	O3	1.701(2)
P2	O5	1.504(2)	Al1	O7[5]	1.715(3)
P2	O6	1.516(2)	O1	Al2[6]	1.681(3)
P2	O7	1.518(3)	O2	Al2[5]	1.716(4)
Al2	O1[2]	1.681(3)	O6	Al1[7]	1.705(2)
Al2	O5[1]	1.722(2)	O7	Al1[3]	1.716(3)

[1]+X,+Y,3/2-Z; [2]+Y-X,1-X,+Z; [3]2-Y,1+X-Y,+Z; [4]+Y,1-X+Y,1-Z; [5]1+Y-X,2-X,+Z; [6]1-Y,1+X-Y,+Z; [7]1-Y+X,+X,1-Z

Table 5 Bond Angles for 17O2_sc2_3p459GPa.

Atom	Atom	Atom	Angle/°	Atom	Atom	Atom	Angle/°
O1	P1	O3[1]	108.29(12)	O2[3]	Al2	O5	109.83(12)
O1	P1	O3	108.29(12)	O2[3]	Al2	O5[1]	109.83(12)
O2	P1	O1	109.8(2)	O4	Al1	O7[4]	110.02(14)
O2	P1	O3[1]	110.50(13)	O6[5]	Al1	O4	109.99(12)
O2	P1	O3	110.50(13)	O6[5]	Al1	O7[4]	108.50(13)
O3[1]	P1	O3	109.4(2)	O3	Al1	O4	109.37(13)
O4	P2	O6	110.80(14)	O3	Al1	O6[5]	111.75(13)
O4	P2	O7	108.16(15)	O3	Al1	O7[4]	107.15(14)
O5	P2	O4	109.73(14)	P1	O1	Al2[6]	179.0(2)
O5	P2	O6	107.78(15)	P2	O4	Al1	148.51(16)
O5	P2	O7	109.70(15)	P2	O5	Al2	139.73(17)
O6	P2	O7	110.67(15)	P1	O2	Al2[4]	161.2(3)
O1[2]	Al2	O5	109.33(11)	P2	O6	Al1[7]	154.0(2)
O1[2]	Al2	O5[1]	109.33(11)	P1	O3	Al1	141.76(17)
O1[2]	Al2	O2[3]	110.39(18)	P2	O7	Al1[3]	148.65(18)
O5	Al2	O5[1]	108.09(19)				

[1]+X,+Y,3/2-Z; [2]+Y-X,1-X,+Z; [3]2-Y,1+X-Y,+Z; [4]1+Y-X,2-X,+Z; [5]+Y,1-X+Y,1-Z; [6]1-Y,1+X-Y,+Z; [7]1-Y+X,+X,1-Z

Table 6 Torsion Angles for 17O2_sc2_3p459GPa.

A	B	C	D	Angle/°	A	B	C	D	Angle/°
O1	P1	O2	Al2[1]	180.0	O6	P2	O5	Al2	141.3(3)
O1	P1	O3	Al1	165.3(3)	O6	P2	O7	Al1[4]	-33.8(4)
O1[2]	Al2	O5	P2	-166.5(3)	O6[6]	Al1	O4	P2	28.3(4)
O4	P2	O5	Al2	-97.9(3)	O6[6]	Al1	O3	P1	-139.2(3)
O4	P2	O6	Al1[3]	70.3(4)	O3[5]	P1	O2	Al2[1]	60.60(12)
O4	P2	O7	Al1[4]	-155.4(3)	O3	P1	O2	Al2[1]	-60.60(12)
O4	Al1	O3	P1	98.8(3)	O3[5]	P1	O3	Al1	-76.8(3)
O5	P2	O4	Al1	-148.8(3)	O3	Al1	O4	P2	151.3(3)
O5	P2	O6	Al1[3]	-169.7(4)	O7	P2	O4	Al1	91.6(3)
O5	P2	O7	Al1[4]	85.0(4)	O7	P2	O5	Al2	20.8(3)
O5[5]	Al2	O5	P2	74.6(3)	O7	P2	O6	Al1[3]	-49.7(4)
O2	P1	O3	Al1	45.0(3)	O7[1]	Al1	O4	P2	-91.2(4)
O2[4]	Al2	O5	P2	-45.2(3)	O7[1]	Al1	O3	P1	-20.5(3)
O6	P2	O4	Al1	-29.9(4)					

[1]1+Y-X,2-X,+Z; [2]+Y-X,1-X,+Z; [3]1-Y+X,+X,1-Z; [4]2-Y,1+X-Y,+Z; [5]+X,+Y,3/2-Z; [6]+Y,1-X+Y,1-Z

Table 7 Solvent masks information for 17O2_sc2_3p459GPa.

Number	X	Y	Z	Volume	Electron count	Content
1	0.000	0.000	0.000	13.4	19.1	?
2	0.000	0.000	0.250	18.0	26.1	?
3	0.000	0.000	0.500	13.4	19.1	?
4	0.000	0.000	0.750	18.0	26.1	?
5	-0.487	-0.061	-0.575	887.4	365.1	?

Experimental

Single crystals of AlO_4P **[17O2_sc2_3p459GPa]** were **[]**. A suitable crystal was selected and **[]** on a **esperanto-CrysAlisPro-abstract goniometer imported esperanto images** diffractometer. The crystal was kept at 293(2) K during data collection. Using Olex2 [1], the structure was solved with the SHELXT [2] structure solution program using Intrinsic Phasing and refined with the SHELXL [3] refinement package using Least Squares minimisation.

1. Dolomanov, O.V., Bourhis, L.J., Gildea, R.J, Howard, J.A.K. & Puschmann, H. (2009), J. Appl. Cryst. 42, 339-341.
2. Sheldrick, G.M. (2015). Acta Cryst. A71, 3-8.
3. Sheldrick, G.M. (2015). Acta Cryst. C71, 3-8.

Crystal structure determination of [17O2_sc2_3p459GPa]

Crystal Data for AlO_4P (M =121.95 g/mol): hexagonal, space group $P6_3/m$ (no. 176), a = 13.0957(6) Å, c = 15.0060(7) Å, V = 2228.7(2) Å^3, Z = 18, T = 293(2) K, μ(synchrotron) = 0.227 mm^{-1}, $Dcalc$ = 1.636 g/cm^3, 2963 reflections measured (4.338° ≤ 2Θ ≤ 31.908°), 1050 unique (R_{int} = 0.0252, R_{sigma} = 0.0344) which were used in all calculations. The final R_1 was 0.0367 (I > 2σ(I)) and wR_2 was 0.1155 (all data).

Refinement model description

Number of restraints - 0, number of constraints - unknown.
Details:
N/A

This report has been created with Olex2, compiled on 2023.03.06 svn.rbb2c1857 for OlexSys. Please let us know if there are any errors or if you would like to have additional features.

17O2_sc2_4p1GPa

Table 1 Crystal data and structure refinement for 17O2_sc2_4p1GPa.

Identification code	17O2_sc2_4p1GPa
Empirical formula	AlO_4P
Formula weight	121.95
Temperature/K	293(2)
Crystal system	hexagonal
Space group	$P6_3/m$
a/Å	13.0773(6)
b/Å	13.0773(6)
c/Å	14.9450(6)
α/°	90
β/°	90
γ/°	120
Volume/Å^3	2213.4(2)
Z	18
ρ_{calc}g/cm^3	1.647
μ/mm^{-1}	0.228
F(000)	1080.0
Crystal size/mm^3	0.125 × 0.050 × 0.050
Radiation	synchrotron (λ = 0.49555)
2Θ range for data collection/°	3.146 to 31.956
Index ranges	$-11 \leq h \leq 10$, $-14 \leq k \leq 14$, $-16 \leq l \leq 16$
Reflections collected	2877
Independent reflections	1051 [R_{int} = 0.0249, R_{sigma} = 0.0350]
Data/restraints/parameters	1051/0/88
Goodness-of-fit on F^2	1.059
Final R indexes [I>=2σ (I)]	R_1 = 0.0375, wR_2 = 0.1099
Final R indexes [all data]	R_1 = 0.0490, wR_2 = 0.1143
Largest diff. peak/hole / e Å^{-3}	0.29/-0.36

Table 2 Fractional Atomic Coordinates (×10^4) and Equivalent Isotropic Displacement Parameters (Å^2×10^3) for 17O2_sc2_4p1GPa. U_{eq} is defined as 1/3 of the trace of the orthogonalised U_{IJ} tensor.

**Atom**	***x***	***y***	***z***	**U(eq)**
P1	6663.5(11)	10911.4(12)	7500	24.4(4)
P2	7612.6(8)	7638.0(8)	6056.9(6)	26.1(3)
Al2	6673.2(12)	5765.6(13)	7500	24.1(4)
Al1	7644.8(9)	10022.3(9)	6042.1(6)	25.7(4)
O1	5525(3)	10922(3)	7500	33.3(9)

Table 2 Fractional Atomic Coordinates (×10⁴) and Equivalent Isotropic Displacement Parameters (Å²×10³) for 17O2_sc2_4p1GPa. U_{eq} is defined as 1/3 of the trace of the orthogonalised U_{IJ} tensor.

Atom	*x*	*y*	*z*	U(eq)
O4	7516(2)	8700(2)	6335.2(15)	35.7(7)
O5	6731(2)	6569(2)	6571.3(16)	39.1(7)
O2	7682(3)	12151(3)	7500	46.4(11)
O6	7346(2)	7376(2)	5071.4(14)	40.2(8)
O3	6701(2)	10257(2)	6672.6(15)	40.8(7)
O7	8851(2)	7892(2)	6269.2(18)	43.5(8)

Table 3 Anisotropic Displacement Parameters (Å²×10³) for 17O2_sc2_4p1GPa. The Anisotropic displacement factor exponent takes the form: $-2\pi^2[h^2a^{*2}U_{11}+2hka^*b^*U_{12}+\ldots]$.

Atom	U_{11}	U_{22}	U_{33}	U_{23}	U_{13}	U_{12}
P1	21.0(8)	26.7(8)	27.5(7)	0	0	13.4(7)
P2	30.4(6)	29.0(6)	21.6(5)	3.2(4)	1.2(4)	17.0(5)
Al2	24.5(9)	20.6(9)	24.1(8)	0	0	8.9(7)
Al1	31.7(7)	25.6(7)	20.2(6)	-1.8(4)	1.6(4)	14.7(6)
O1	28(2)	33(2)	40(2)	0	0	15.5(18)
O4	46.2(18)	30.2(16)	34.8(13)	3.6(11)	4.1(11)	22.2(14)
O5	51.8(18)	34.7(16)	26.9(12)	7.7(10)	5.3(11)	18.7(15)
O2	27(2)	35(2)	74(3)	0	0	13(2)
O6	51.3(18)	53.2(19)	19.0(13)	1.2(11)	0.4(11)	28.2(16)
O3	42.4(18)	50.4(18)	35.0(14)	-4.2(13)	7.1(12)	27.3(15)
O7	37.2(17)	49.8(19)	48.4(16)	-7.8(13)	-3.8(12)	25.6(15)

Table 4 Bond Lengths for 17O2_sc2_4p1GPa.

Atom	Atom	Length/Å	Atom	Atom	Length/Å
P1	O1	1.495(3)	Al2	O1[2]	1.679(3)
P1	O2	1.498(4)	Al2	O5[1]	1.719(2)
P1	O3	1.519(2)	Al2	O5	1.719(2)
P1	O3[1]	1.519(2)	Al2	O2[3]	1.712(4)
P2	O4	1.514(2)	Al1	O4	1.708(2)
P2	O5	1.504(3)	Al1	O6[4]	1.702(2)
P2	O6	1.513(2)	Al1	O3	1.698(2)
P2	O7	1.515(3)	Al1	O7[5]	1.712(3)

[1]+X,+Y,3/2-Z; [2]+Y-X,1-X,+Z; [3]2-Y,1+X-Y,+Z; [4]+Y,1-X+Y,1-Z; [5]1+Y-X,2-X,+Z

Table 5 Bond Angles for 17O2_sc2_4p1GPa.

Atom	Atom	Atom	Angle/°	Atom	Atom	Atom	Angle/°
O1	P1	O2	109.9(2)	O2[3]	Al2	O5[1]	109.88(12)
O1	P1	O3	108.53(13)	O2[3]	Al2	O5	109.88(12)
O1	P1	O3[1]	108.53(13)	O4	Al1	O7[4]	110.08(14)
O2	P1	O3[1]	110.39(14)	O6[5]	Al1	O4	109.91(13)
O2	P1	O3	110.39(14)	O6[5]	Al1	O7[4]	108.88(14)
O3	P1	O3[1]	109.0(2)	O3	Al1	O4	109.13(13)
O4	P2	O7	108.16(15)	O3	Al1	O6[5]	111.74(14)
O5	P2	O4	109.62(14)	O3	Al1	O7[4]	107.07(14)
O5	P2	O6	107.71(15)	P1	O1	Al2[6]	179.0(2)
O5	P2	O7	109.57(15)	P2	O4	Al1	147.84(17)
O6	P2	O4	111.07(14)	P2	O5	Al2	139.46(18)
O6	P2	O7	110.70(15)	P1	O2	Al2[4]	161.4(3)
O1[2]	Al2	O5[1]	109.41(12)	P2	O6	Al1[7]	154.7(2)
O1[2]	Al2	O5	109.41(12)	P1	O3	Al1	141.70(18)
O1[2]	Al2	O2[3]	110.51(19)	P2	O7	Al1[3]	148.10(18)
O5[1]	Al2	O5	107.69(19)				

[1]+X,+Y,3/2-Z; [2]+Y-X,1-X,+Z; [3]2-Y,1+X-Y,+Z; [4]1+Y-X,2-X,+Z; [5]+Y,1-X+Y,1-Z; [6]1-Y,1+X-Y,+Z; [7]1-Y+X,+X,1-Z

Table 6 Torsion Angles for 17O2_sc2_4p1GPa.

A	B	C	D	Angle/°	A	B	C	D	Angle/°
O1	P1	O2	Al2[1]	180.000(0)	O6	P2	O5	Al2	141.1(3)
O1	P1	O3	Al1	165.5(3)	O6	P2	O7	Al1[4]	-32.9(4)
O1[2]	Al2	O5	P2	-166.7(3)	O6[6]	Al1	O4	P2	28.7(4)
O4	P2	O5	Al2	-98.0(3)	O6[6]	Al1	O3	P1	-139.4(3)
O4	P2	O6	Al1[3]	69.5(4)	O3	P1	O2	Al2[1]	-60.31(13)
O4	P2	O7	Al1[4]	-154.8(3)	O3[5]	P1	O2	Al2[1]	60.31(13)
O4	Al1	O3	P1	98.8(3)	O3[5]	P1	O3	Al1	-76.5(4)
O5	P2	O4	Al1	-149.0(3)	O3	Al1	O4	P2	151.6(3)
O5	P2	O6	Al1[3]	-170.4(4)	O7	P2	O4	Al1	91.6(3)
O5	P2	O7	Al1[4]	85.8(4)	O7	P2	O5	Al2	20.6(3)
O5[5]	Al2	O5	P2	74.5(3)	O7	P2	O6	Al1[3]	-50.6(4)
O2	P1	O3	Al1	45.0(3)	O7[1]	Al1	O4	P2	-91.2(3)
O2[4]	Al2	O5	P2	-45.2(3)	O7[1]	Al1	O3	P1	-20.3(3)
O6	P2	O4	Al1	-30.1(4)					

[1]1+Y-X,2-X,+Z; [2]+Y-X,1-X,+Z; [3]1-Y+X,+X,1-Z; [4]2-Y,1+X-Y,+Z; [5]+X,+Y,3/2-Z; [6]+Y,1-X+Y,1-Z

Table 7 Solvent masks information for 17O2_sc2_4p1GPa.

Number	X	Y	Z	Volume	Electron count	Content
1	0.000	0.000	0.000	13.3	20.1	?
2	0.000	0.000	0.250	17.9	27.3	?
3	0.000	0.000	0.500	13.3	20.1	?
4	0.000	0.000	0.750	17.9	27.3	?
5	-0.163	0.582	0.510	880.2	383.2	?

Experimental

Single crystals of AlO_4P **[17O2_sc2_4p1GPa]** were **[]**. A suitable crystal was selected and **[]** on a **esperanto-CrysAlisPro-abstract goniometer imported esperanto images** diffractometer. The crystal was kept at 293(2) K during data collection. Using Olex2 [1], the structure was solved with the SHELXT [2] structure solution program using Intrinsic Phasing and refined with the SHELXL [3] refinement package using Least Squares minimisation.

1. Dolomanov, O.V., Bourhis, L.J., Gildea, R.J, Howard, J.A.K. & Puschmann, H. (2009), J. Appl. Cryst. 42, 339-341.
2. Sheldrick, G.M. (2015). Acta Cryst. A71, 3-8.
3. Sheldrick, G.M. (2015). Acta Cryst. C71, 3-8.

Crystal structure determination of [17O2_sc2_4p1GPa]

Crystal Data for AlO_4P (M =121.95 g/mol): hexagonal, space group $P6_3/m$ (no. 176), a = 13.0773(6) Å, c = 14.9450(6) Å, V = 2213.4(2) Å^3, Z = 18, T = 293(2) K, μ(synchrotron) = 0.228 mm^{-1}, $Dcalc$ = 1.647 g/cm^3, 2877 reflections measured (3.146° ≤ 2Θ ≤ 31.956°), 1051 unique (R_{int} = 0.0249, R_{sigma} = 0.0350) which were used in all calculations. The final R_1 was 0.0375 (I > 2σ(I)) and wR_2 was 0.1143 (all data).

Refinement model description

Number of restraints - 0, number of constraints - unknown.

Details:

N/A

This report has been created with Olex2, compiled on 2023.03.06 svn.rbb2c1857 for OlexSys. Please let us know if there are any errors or if you would like to have additional features.

$AlPO_4$-17/O_2 4.6GPa

Table 1 Crystal data and structure refinement for 17O2_sc2_4p6GPa.

Identification code	17O2_sc2_4p6GPa
Empirical formula	AlO_4P
Formula weight	121.95
Temperature/K	293(2)
Crystal system	hexagonal
Space group	$P6_3/m$
a/Å	13.0545(8)
b/Å	13.0545(8)
c/Å	14.9154(7)
α/°	90
β/°	90
γ/°	120
Volume/Å^3	2201.3(3)
Z	18
ρ_{calc}g/cm^3	1.656
μ/mm^{-1}	0.230
F(000)	1080.0
Crystal size/mm^3	0.125 × 0.050 × 0.050
Radiation	synchrotron (λ = 0.49555)
2Θ range for data collection/°	3.152 to 31.958
Index ranges	$-14 \le h \le 14$, $-11 \le k \le 11$, $-16 \le l \le 15$
Reflections collected	2894
Independent reflections	1048 [R_{int} = 0.0270, R_{sigma} = 0.0383]
Data/restraints/parameters	1048/0/88
Goodness-of-fit on F^2	1.062
Final R indexes [I>=2σ (I)]	R_1 = 0.0448, wR_2 = 0.1211
Final R indexes [all data]	R_1 = 0.0593, wR_2 = 0.1272
Largest diff. peak/hole / e Å^{-3}	0.27/-0.35

Table 2 Fractional Atomic Coordinates (×10^4) and Equivalent Isotropic Displacement Parameters (Å^2×10^3) for 17O2_sc2_4p6GPa. U_{eq} is defined as 1/3 of the trace of the orthogonalised U_{IJ} tensor.

Atom	*x*	*y*	*z*	U(eq)
P1	6662.8(14)	10913.5(15)	7500	30.1(5)
P2	7613.4(10)	7638.3(10)	6058.9(7)	30.1(4)
Al2	6672.0(16)	5762.9(16)	7500	29.5(5)
Al1	7646.5(12)	10021.3(11)	6043.2(8)	30.1(4)
O1	5523(4)	10921(4)	7500	39.2(11)

Table 2 Fractional Atomic Coordinates (×10^4) and Equivalent Isotropic Displacement Parameters (Å^2×10^3) for 17O2_sc2_4p6GPa. U_{eq} is defined as 1/3 of the trace of the orthogonalised U_{IJ} tensor.

Atom	*x*	*y*	*z*	U(eq)
O4	7515(3)	8698(3)	6336.8(19)	40.2(8)
O5	6730(3)	6570(3)	6574(2)	45.2(9)
O2	7677(4)	12153(4)	7500	59.6(15)
O6	7342(3)	7374(3)	5072.0(18)	45.9(9)
O3	6698(3)	10255(3)	6674(2)	48.5(9)
O7	8856(3)	7897(3)	6271(2)	51.1(10)

Table 3 Anisotropic Displacement Parameters (Å^2×10^3) for 17O2_sc2_4p6GPa. The Anisotropic displacement factor exponent takes the form: -2π^2[h^{2}a*^{2}U$_{11}$+2hka*b*U$_{12}$+…].

Atom	U_{11}	U_{22}	U_{33}	U_{23}	U_{13}	U_{12}
P1	25.1(10)	34.7(10)	32.6(9)	0	0	16.5(9)
P2	36.1(8)	34.8(8)	22.9(6)	4.1(5)	2.6(5)	20.5(6)
Al2	29.9(12)	26.3(11)	28.8(9)	0	0	11.4(9)
Al1	36.7(9)	30.4(9)	22.9(7)	-0.9(5)	3.4(5)	16.6(7)
O1	39(3)	42(3)	37(2)	0	0	21(2)
O4	54(2)	40(2)	34.1(16)	1.7(14)	2.8(14)	28.6(18)
O5	59(2)	43(2)	31.8(16)	9.3(14)	8.8(14)	24.1(18)
O2	35(3)	42(3)	96(4)	0	0	15(3)
O6	57(2)	60(2)	22.5(17)	1.4(14)	3.2(14)	31(2)
O3	52(2)	58(2)	40.4(19)	-2.9(16)	9.5(15)	31.5(19)
O7	47(2)	61(2)	55(2)	-6.1(17)	-1.8(16)	34(2)

Table 4 Bond Lengths for 17O2_sc2_4p6GPa.

Atom	Atom	Length/Å	Atom	Atom	Length/Å
P1	O1	1.493(4)	Al2	O5	1.716(3)
P1	O2	1.493(5)	Al2	O2[3]	1.711(5)
P1	O3[1]	1.516(3)	Al1	O4	1.706(3)
P1	O3	1.516(3)	Al1	O6[4]	1.702(3)
P2	O4	1.510(3)	Al1	O3	1.700(3)
P2	O5	1.502(3)	Al1	O7[5]	1.706(3)
P2	O6	1.513(3)	O1	Al2[6]	1.670(4)
P2	O7	1.516(3)	O2	Al2[5]	1.711(5)
Al2	O1[2]	1.670(4)	O6	Al1[7]	1.702(3)
Al2	O5[1]	1.716(3)	O7	Al1[3]	1.706(3)

[1]+X,+Y,3/2-Z; [2]+Y-X,1-X,+Z; [3]2-Y,1+X-Y,+Z; [4]+Y,1-X+Y,1-Z; [5]1+Y-X,2-X,+Z; [6]1-Y,1+X-Y,+Z; [7]1-Y+X,+X,1-Z

Table 5 Bond Angles for 17O2_sc2_4p6GPa.

Atom	Atom	Atom	Angle/°	Atom	Atom	Atom	Angle/°
O1	P1	O3	108.45(16)	O2[3]	Al2	O5[1]	110.11(16)
O1	P1	O3[1]	108.45(16)	O2[3]	Al2	O5	110.11(16)
O2	P1	O1	109.8(3)	O4	Al1	O7[4]	110.05(18)
O2	P1	O3[1]	110.66(18)	O6[5]	Al1	O4	110.09(16)
O2	P1	O3	110.66(18)	O6[5]	Al1	O7[4]	108.97(17)
O3	P1	O3[1]	108.7(3)	O3	Al1	O4	109.06(17)
O4	P2	O6	111.00(17)	O3	Al1	O6[5]	111.42(18)
O4	P2	O7	108.08(19)	O3	Al1	O7[4]	107.21(18)
O5	P2	O4	109.43(18)	P1	O1	Al2[6]	179.2(3)
O5	P2	O6	107.6(2)	P2	O4	Al1	147.8(2)
O5	P2	O7	109.85(19)	P2	O5	Al2	139.4(2)
O6	P2	O7	110.87(19)	P1	O2	Al2[4]	161.1(4)
O1[2]	Al2	O5[1]	109.45(15)	P2	O6	Al1[7]	154.4(3)
O1[2]	Al2	O5	109.45(15)	P1	O3	Al1	141.5(2)
O1[2]	Al2	O2[3]	110.4(2)	P2	O7	Al1[3]	147.9(2)
O5[1]	Al2	O5	107.2(2)				

[1]+X,+Y,3/2-Z; [2]+Y-X,1-X,+Z; [3]2-Y,1+X-Y,+Z; [4]1+Y-X,2-X,+Z; [5]+Y,1-X+Y,1-Z; [6]1-Y,1+X-Y,+Z; [7]1-Y+X,+X,1-Z

Table 6 Torsion Angles for 17O2_sc2_4p6GPa.

A	B	C	D	Angle/°	A	B	C	D	Angle/°
O1	P1	O2	Al2[1]	180.000(1)	O6	P2	O5	Al2	140.8(3)
O1	P1	O3	Al1	165.5(4)	O6	P2	O7	Al1[4]	-33.1(5)
O1[2]	Al2	O5	P2	-166.4(3)	O6[6]	Al1	O4	P2	29.1(5)
O4	P2	O5	Al2	-98.5(3)	O6[6]	Al1	O3	P1	-139.1(4)
O4	P2	O6	Al1[3]	70.2(5)	O3[5]	P1	O2	Al2[1]	60.30(17)
O4	P2	O7	Al1[4]	-155.0(4)	O3	P1	O2	Al2[1]	-60.30(17)
O4	Al1	O3	P1	99.2(4)	O3[5]	P1	O3	Al1	-76.8(5)
O5	P2	O4	Al1	-149.2(4)	O3	Al1	O4	P2	151.6(4)
O5	P2	O6	Al1[3]	-170.1(5)	O7	P2	O4	Al1	91.2(4)
O5	P2	O7	Al1[4]	85.6(5)	O7	P2	O5	Al2	20.0(4)
O5[5]	Al2	O5	P2	75.0(4)	O7	P2	O6	Al1[3]	-50.0(5)
O2	P1	O3	Al1	45.0(4)	O7[1]	Al1	O4	P2	-91.0(4)
O2[4]	Al2	O5	P2	-44.8(4)	O7[1]	Al1	O3	P1	-20.0(4)
O6	P2	O4	Al1	-30.6(5)					

[1]1+Y-X,2-X,+Z; [2]+Y-X,1-X,+Z; [3]1-Y+X,+X,1-Z; [4]2-Y,1+X-Y,+Z; [5]+X,+Y,3/2-Z; [6]+Y,1-X+Y,1-Z

Table 7 Solvent masks information for 17O2_sc2_4p6GPa.

Number	X	Y	Z	Volume	Electron count	Content
1	0.000	0.000	0.000	13.3	20.9	?
2	0.000	0.000	0.250	17.8	27.2	?
3	0.000	0.000	0.500	13.3	20.9	?
4	0.000	0.000	0.750	17.8	27.2	?
5	-0.463	0.110	-0.447	869.4	407.4	?

Experimental

Single crystals of AlO_4P **[17O2_sc2_4p6GPa]** were **[]**. A suitable crystal was selected and **[]** on a **esperanto-CrysAlisPro-abstract goniometer imported esperanto images** diffractometer. The crystal was kept at 293(2) K during data collection. Using Olex2 [1], the structure was solved with the SHELXT [2] structure solution program using Intrinsic Phasing and refined with the SHELXL [3] refinement package using Least Squares minimisation.

1. Dolomanov, O.V., Bourhis, L.J., Gildea, R.J, Howard, J.A.K. & Puschmann, H. (2009), J. Appl. Cryst. 42, 339-341.
2. Sheldrick, G.M. (2015). Acta Cryst. A71, 3-8.
3. Sheldrick, G.M. (2015). Acta Cryst. C71, 3-8.

Crystal structure determination of [17O2_sc2_4p6GPa]

Crystal Data for AlO_4P (M =121.95 g/mol): hexagonal, space group $P6_3/m$ (no. 176), a = 13.0545(8) Å, c = 14.9154(7) Å, V = 2201.3(3) Å^3, Z = 18, T = 293(2) K, μ(synchrotron) = 0.230 mm^{-1}, $Dcalc$ = 1.656 g/cm^3, 2894 reflections measured (3.152° ≤ 2Θ ≤ 31.958°), 1048 unique (R_{int} = 0.0270, R_{sigma} = 0.0383) which were used in all calculations. The final R_1 was 0.0448 (I > 2σ(I)) and wR_2 was 0.1272 (all data).

Refinement model description

Number of restraints - 0, number of constraints - unknown.
Details:
N/A

This report has been created with Olex2, compiled on 2023.03.06 svn.rbb2c1857 for OlexSys. Please let us know if there are any errors or if you would like to have additional features.

$AlPO_4$-17/O_2 5.46GPa

Table 1 Crystal data and structure refinement for 17O2_sc2_5p46GPa.

Identification code	17O2_sc2_5p46GPa
Empirical formula	AlO_4P
Formula weight	121.95
Temperature/K	293(2)
Crystal system	hexagonal
Space group	$P6_3/m$
a/Å	13.0486(9)
b/Å	13.0486(9)
c/Å	14.7950(10)
α/°	90
β/°	90
γ/°	120
Volume/Å^3	2181.6(3)
Z	18
ρ_{calc}g/cm^3	1.671
μ/mm^{-1}	0.232
F(000)	1080.0
Crystal size/mm^3	0.125 × 0.050 × 0.050
Radiation	synchrotron (λ = 0.49555)
2Θ range for data collection/°	3.162 to 31.954
Index ranges	$-12 \leq h \leq 9$, $-14 \leq k \leq 14$, $-16 \leq l \leq 15$
Reflections collected	2755
Independent reflections	1042 [R_{int} = 0.0172, R_{sigma} = 0.0213]
Data/restraints/parameters	1042/0/88
Goodness-of-fit on F^2	1.047
Final R indexes [I>=2σ (I)]	R_1 = 0.0450, wR_2 = 0.1339
Final R indexes [all data]	R_1 = 0.0537, wR_2 = 0.1393
Largest diff. peak/hole / e Å^{-3}	0.31/-0.36

Table 2 Fractional Atomic Coordinates (×10^4) and Equivalent Isotropic Displacement Parameters (Å^2×10^3) for 17O2_sc2_5p46GPa. U_{eq} is defined as 1/3 of the trace of the orthogonalised U_{IJ} tensor.

Atom	*x*	*y*	*z*	U(eq)
P1	6662.2(13)	10914.8(13)	7500	37.6(5)
P2	7619.6(10)	7645.5(10)	6067.9(7)	39.2(4)
Al2	6673.9(14)	5761.9(14)	7500	36.4(5)
Al1	7652.3(11)	10020.5(11)	6050.7(8)	38.8(4)
O1	5523(3)	10918(4)	7500	49.1(11)

Table 2 Fractional Atomic Coordinates (×10^4) and Equivalent Isotropic Displacement Parameters (Å^2×10^3) for 17O2_sc2_5p46GPa. U_{eq} is defined as 1/3 of the trace of the orthogonalised U_{IJ} tensor.

Atom	*x*	*y*	*z*	U(eq)
O4	7523(3)	8702(3)	6355.6(19)	50.5(8)
O5	6724(3)	6564(3)	6564.9(18)	54.9(9)
O2	7678(4)	12156(4)	7500	63.3(13)
O6	7377(3)	7407(3)	5071.1(17)	57.4(10)
O3	6694(3)	10256(3)	6668.8(18)	55.9(9)
O7	8852(3)	7887(3)	6293(2)	58.8(9)

Table 3 Anisotropic Displacement Parameters (Å^2×10^3) for 17O2_sc2_5p46GPa. The Anisotropic displacement factor exponent takes the form: -2π^2[h^{2}a*^{2}U_{11}+2hka*b*U_{12}+…].

Atom	U_{11}	U_{22}	U_{33}	U_{23}	U_{13}	U_{12}
P1	35.2(9)	41.2(10)	39.2(8)	0	0	21.3(8)
P2	48.6(7)	44.8(7)	29.0(6)	5.1(4)	2.4(4)	26.9(6)
Al2	36.4(11)	34.0(10)	34.4(9)	0	0	14.2(8)
Al1	48.2(8)	40.9(8)	27.2(7)	-1.5(5)	3.0(5)	22.2(6)
O1	45(3)	51(3)	52(2)	0	0	25(2)
O4	66(2)	50.0(19)	43.4(16)	3.3(13)	4.3(14)	34.5(17)
O5	67(2)	50.9(19)	39.0(16)	8.2(13)	4.8(14)	23.6(17)
O2	40(3)	47(3)	102(4)	0	0	20(2)
O6	76(2)	76(2)	28.9(17)	4.4(13)	1.3(13)	44(2)
O3	60(2)	68(2)	47.0(17)	-2.8(15)	7.0(14)	37.7(18)
O7	55(2)	65(2)	61.2(19)	-6.1(16)	-2.6(15)	34.4(18)

Table 4 Bond Lengths for 17O2_sc2_5p46GPa.

Atom	Atom	Length/Å	Atom	Atom	Length/Å
P1	O1	1.488(4)	Al2	O5	1.716(3)
P1	O2	1.494(5)	Al2	O2^3	1.706(5)
P1	O3^1	1.513(3)	Al1	O4	1.703(3)
P1	O3	1.513(3)	Al1	O6^4	1.692(3)
P2	O4	1.508(3)	Al1	O3	1.697(3)
P2	O5	1.500(3)	Al1	O7^5	1.701(3)
P2	O6	1.508(3)	O1	Al2^6	1.673(4)
P2	O7	1.513(3)	O2	Al2^5	1.706(5)
Al2	O1^2	1.673(4)	O6	Al1^7	1.692(3)
Al2	O5^1	1.716(3)	O7	Al1^3	1.701(3)

^{1}+X,+Y,3/2-Z; ^{2}+Y-X,1-X,+Z; ^{3}2-Y,1+X-Y,+Z; ^{4}+Y,1-X+Y,1-Z; ^{5}1+Y-X,2-X,+Z; ^{6}1-Y,1+X-Y,+Z; ^{7}1-Y+X,+X,1-Z

Table 5 Bond Angles for 17O2_sc2_5p46GPa.

Atom	Atom	Atom	Angle/°	Atom	Atom	Atom	Angle/°
O1	P1	O2	110.0(2)	O2[3]	Al2	O5[1]	110.32(15)
O1	P1	O3[1]	108.23(15)	O2[3]	Al2	O5	110.32(15)
O1	P1	O3	108.22(15)	O6[4]	Al1	O4	110.10(16)
O2	P1	O3	110.75(16)	O6[4]	Al1	O3	111.61(17)
O2	P1	O3[1]	110.74(16)	O6[4]	Al1	O7[5]	108.83(17)
O3	P1	O3[1]	108.8(3)	O3	Al1	O4	109.30(16)
O4	P2	O7	108.30(18)	O3	Al1	O7[5]	106.79(17)
O5	P2	O4	109.89(18)	O7[5]	Al1	O4	110.15(17)
O5	P2	O6	107.52(19)	P1	O1	Al2[6]	179.6(3)
O5	P2	O7	109.58(18)	P2	O4	Al1	146.9(2)
O6	P2	O4	111.10(17)	P2	O5	Al2	138.0(2)
O6	P2	O7	110.45(19)	P1	O2	Al2[5]	161.0(3)
O1[2]	Al2	O5	109.03(13)	P2	O6	Al1[7]	156.9(3)
O1[2]	Al2	O5[1]	109.03(14)	P1	O3	Al1	140.6(2)
O1[2]	Al2	O2[3]	110.6(2)	P2	O7	Al1[3]	147.8(2)
O5	Al2	O5[1]	107.5(2)				

[1]+X,+Y,3/2-Z; [2]+Y-X,1-X,+Z; [3]2-Y,1+X-Y,+Z; [4]+Y,1-X+Y,1-Z; [5]1+Y-X,2-X,+Z; [6]1-Y,1+X-Y,+Z; [7]1-Y+X,+X,1-Z

Table 6 Torsion Angles for 17O2_sc2_5p46GPa.

A	B	C	D	Angle/°	A	B	C	D	Angle/°
O1	P1	O2	Al2[1]	180.000(1)	O6	P2	O5	Al2	141.5(3)
O1	P1	O3	Al1	166.7(3)	O6	P2	O7	Al1[4]	-30.7(5)
O1[2]	Al2	O5	P2	-168.0(3)	O6[6]	Al1	O4	P2	27.3(4)
O4	P2	O5	Al2	-97.5(3)	O6[6]	Al1	O3	P1	-139.7(3)
O4	P2	O6	Al1[3]	69.5(6)	O3[5]	P1	O2	Al2[1]	60.39(15)
O4	P2	O7	Al1[4]	-152.6(4)	O3	P1	O2	Al2[1]	-60.39(15)
O4	Al1	O3	P1	98.2(4)	O3[5]	P1	O3	Al1	-75.9(4)
O5	P2	O4	Al1	-147.4(4)	O3	Al1	O4	P2	150.3(4)
O5	P2	O6	Al1[3]	-170.3(5)	O7	P2	O4	Al1	92.9(4)
O5	P2	O7	Al1[4]	87.5(5)	O7	P2	O5	Al2	21.4(4)
O5[5]	Al2	O5	P2	74.0(4)	O7	P2	O6	Al1[3]	-50.7(6)
O2	P1	O3	Al1	46.0(4)	O7[1]	Al1	O4	P2	-92.7(4)
O2[4]	Al2	O5	P2	-46.3(4)	O7[1]	Al1	O3	P1	-20.9(4)
O6	P2	O4	Al1	-28.5(4)					

[1]1+Y-X,2-X,+Z; [2]+Y-X,1-X,+Z; [3]1-Y+X,+X,1-Z; [4]2-Y,1+X-Y,+Z; [5]+X,+Y,3/2-Z; [6]+Y,1-X+Y,1-Z

Table 7 Solvent masks information for 17O2_sc2_5p46GPa.

Number	X	Y	Z	Volume	Electron count	Content
1	0.000	0.000	0.000	13.3	21.5	?
2	0.000	0.000	0.250	16.4	27.3	?
3	0.000	0.000	0.500	13.3	21.5	?
4	0.000	0.000	0.750	16.4	27.3	?
5	-0.850	-0.298	-0.132	864.0	426.1	?

Experimental

Single crystals of AlO_4P **[17O2_sc2_5p46GPa]** were **[]**. A suitable crystal was selected and **[]** on a **esperanto-CrysAlisPro-abstract goniometer imported esperanto images** diffractometer. The crystal was kept at 293(2) K during data collection. Using Olex2 [1], the structure was solved with the SHELXT [2] structure solution program using Intrinsic Phasing and refined with the SHELXL [3] refinement package using Least Squares minimisation.

1. Dolomanov, O.V., Bourhis, L.J., Gildea, R.J, Howard, J.A.K. & Puschmann, H. (2009), J. Appl. Cryst. 42, 339-341.
2. Sheldrick, G.M. (2015). Acta Cryst. A71, 3-8.
3. Sheldrick, G.M. (2015). Acta Cryst. C71, 3-8.

Crystal structure determination of [17O2_sc2_5p46GPa]

Crystal Data for AlO_4P (M =121.95 g/mol): hexagonal, space group $P6_3/m$ (no. 176), a = 13.0486(9) Å, c = 14.7950(10) Å, V = 2181.6(3) Å^3, Z = 18, T = 293(2) K, μ(synchrotron) = 0.232 mm^{-1}, $Dcalc$ = 1.671 g/cm^3, 2755 reflections measured (3.162° ≤ 2Θ ≤ 31.954°), 1042 unique (R_{int} = 0.0172, R_{sigma} = 0.0213) which were used in all calculations. The final R_1 was 0.0450 (I > 2σ(I)) and wR_2 was 0.1393 (all data).

Refinement model description

Number of restraints - 0, number of constraints - unknown.
Details:
N/A

This report has been created with Olex2, compiled on 2023.03.06 svn.rbb2c1857 for OlexSys. Please let us know if there are any errors or if you would like to have additional features.

$AlPO_4$-17/O_2 0.43GPa

Table 1 Crystal data and structure refinement for 17O2_sc2_0p43GPa.

Identification code	17O2_sc2_0p43GPa
Empirical formula	AlO_4P
Formula weight	121.95
Temperature/K	293(2)
Crystal system	hexagonal
Space group	$P6_3/m$
a/Å	13.0719(5)
b/Å	13.0719(5)
c/Å	15.3402(6)
α/°	90
β/°	90
γ/°	120
Volume/Å^3	2270.1(2)
Z	18
ρ_{calc}g/cm^3	1.606
μ/mm^{-1}	0.223
F(000)	1080.0
Crystal size/mm^3	0.125 × 0.050 × 0.050
Radiation	synchrotron (λ = 0.49555)
2Θ range for data collection/°	3.118 to 31.956
Index ranges	$-11 \leq h \leq 11$, $-14 \leq k \leq 14$, $-16 \leq l \leq 16$
Reflections collected	2825
Independent reflections	1091 [R_{int} = 0.0200, R_{sigma} = 0.0249]
Data/restraints/parameters	1091/0/88
Goodness-of-fit on F^2	1.080
Final R indexes [I>=2σ (I)]	R_1 = 0.0332, wR_2 = 0.0993
Final R indexes [all data]	R_1 = 0.0406, wR_2 = 0.1041
Largest diff. peak/hole / e Å^{-3}	0.30/-0.46

Table 2 Fractional Atomic Coordinates (×10^4) and Equivalent Isotropic Displacement Parameters (Å^2×10^3) for 17O2_sc2_0p43GPa. U_{eq} is defined as 1/3 of the trace of the orthogonalised U_{IJ} tensor.

Atom	*x*	*y*	*z*	U(eq)
P1	6688.5(11)	10921.5(12)	7500	29.9(4)
P2	7631.1(8)	7605.5(8)	6002.3(6)	31.3(3)
Al2	6670.6(10)	5748.6(10)	7500	11.0(3)
Al1	7597.2(8)	9973.3(7)	6006.8(5)	13.5(3)
O1	5343(3)	10813(3)	7500	29.2(8)

Table 2 Fractional Atomic Coordinates (×10^4) and Equivalent Isotropic Displacement Parameters (Å^2×10^3) for 17O2_sc2_0p43GPa. U_{eq} is defined as 1/3 of the trace of the orthogonalised U_{IJ} tensor.

Atom	*x*	*y*	*z*	U(eq)
O4	7464(2)	8789(2)	6252.4(15)	33.8(6)
O5	6780(2)	6466(2)	6691.6(15)	35.8(7)
O2	7790(3)	12386(3)	7500	37.5(9)
O6	7257(2)	7214(2)	4930.0(14)	34.6(6)
O3	6828(2)	10232(2)	6593.5(15)	33.4(6)
O7	9085(2)	7981(2)	6121.7(16)	39.3(7)

Table 3 Anisotropic Displacement Parameters (Å^2×10^3) for 17O2_sc2_0p43GPa. The Anisotropic displacement factor exponent takes the form: -2π^2[h^{2}a*^{2}U$_{11}$+2hka*b*U$_{12}$+…].

Atom	U_{11}	U_{22}	U_{33}	U_{23}	U_{13}	U_{12}
P1	26.5(8)	30.6(8)	34.2(8)	0	0	15.5(6)
P2	34.1(6)	30.7(6)	31.1(6)	2.8(4)	2.4(4)	17.7(5)
Al2	8.4(7)	6.4(7)	15.8(6)	0	0	1.8(5)
Al1	15.3(5)	10.3(5)	15.2(5)	-0.3(3)	2.4(4)	6.6(4)
O1	20.8(18)	35(2)	37.2(18)	0	0	18.0(16)
O4	36.1(14)	28.5(14)	41.4(14)	3.4(11)	8.0(11)	19.5(12)
O5	35.5(15)	29.7(14)	36.0(15)	8.8(11)	4.7(11)	11.7(12)
O2	30(2)	26(2)	55(2)	0	0	12.4(17)
O6	37.6(15)	40.5(15)	24.0(13)	1.6(10)	-0.9(10)	18.2(13)
O3	39.2(15)	40.6(15)	29.1(13)	-1.8(11)	4.9(10)	26.5(13)
O7	28.7(14)	39.7(15)	51.6(16)	-5.0(13)	-3.1(12)	18.7(12)

Table 4 Bond Lengths for 17O2_sc2_0p43GPa.

Atom	Atom	Length/Å	Atom	Atom	Length/Å
P1	O1	1.692(3)	Al2	O5	1.518(2)
P1	O2	1.727(3)	Al2	O2^3	1.510(4)
P1	O3^1	1.715(2)	Al1	O4	1.516(2)
P1	O3	1.715(2)	Al1	O6^4	1.519(2)
P2	O4	1.711(2)	Al1	O3	1.509(2)
P2	O5	1.708(2)	Al1	O7^5	1.530(2)
P2	O6	1.719(2)	O1	Al2^6	1.504(3)
P2	O7	1.718(2)	O2	Al2^5	1.510(4)
Al2	O1^2	1.504(3)	O6	Al1^7	1.519(2)
Al2	O5^1	1.518(2)	O7	Al1^3	1.530(2)

^{1}+X,+Y,3/2-Z; ^{2}+Y-X,1-X,+Z; ^{3}2-Y,1+X-Y,+Z; ^{4}+Y,1-X+Y,1-Z; ^{5}1+Y-X,2-X,+Z; ^{6}1-Y,1+X-Y,+Z; ^{7}1-Y+X,+X,1-Z

Table 5 Bond Angles for 17O2_sc2_0p43GPa.

Atom	Atom	Atom	Angle/°	Atom	Atom	Atom	Angle/°
O1	P1	O2	110.43(17)	O2^{3}	Al2	O5	110.22(12)
O1	P1	O3	109.55(11)	O2^{3}	Al2	O5^{1}	110.22(12)
O1	P1	O3^{1}	109.55(11)	O4	Al1	O6^{4}	110.61(14)
O3	P1	O2	109.47(11)	O4	Al1	O7^{5}	107.94(14)
O3^{1}	P1	O2	109.47(11)	O6^{4}	Al1	O7^{5}	110.24(14)
O3	P1	O3^{1}	108.32(18)	O3	Al1	O4	109.37(13)
O4	P2	O6	109.74(12)	O3	Al1	O6^{4}	108.37(14)
O4	P2	O7	109.93(13)	O3	Al1	O7^{5}	110.30(14)
O5	P2	O4	109.11(13)	Al2^{6}	O1	P1	171.1(2)
O5	P2	O6	111.61(13)	Al1	O4	P2	150.01(16)
O5	P2	O7	109.18(13)	Al2	O5	P2	149.74(17)
O7	P2	O6	107.25(13)	Al2^{5}	O2	P1	151.3(2)
O1^{2}	Al2	O5	108.52(12)	Al1^{7}	O6	P2	144.19(18)
O1^{2}	Al2	O5^{1}	108.52(12)	Al1	O3	P1	149.22(17)
O1^{2}	Al2	O2^{3}	109.77(19)	Al1^{3}	O7	P2	147.60(18)
O5	Al2	O5^{1}	109.5(2)				

^{1}+X,+Y,3/2-Z; ^{2}+Y-X,1-X,+Z; ^{3}2-Y,1+X-Y,+Z; ^{4}+Y,1-X+Y,1-Z; ^{5}1+Y-X,2-X,+Z; ^{6}1-Y,1+X-Y,+Z; ^{7}1-Y+X,+X,1-Z

Table 6 Torsion Angles for 17O2_sc2_0p43GPa.

A	B	C	D	Angle/°	A	B	C	D	Angle/°
O1	P1	O2	Al2^{1}	180.0	O6	P2	O5	Al2	139.1(3)
O1	P1	O3	Al1	170.0(3)	O6	P2	O7	Al1^{4}	-54.3(4)
O1^{2}	Al2	O5	P2	-168.9(3)	O6^{6}	Al1	O4	P2	43.5(4)
O4	P2	O5	Al2	-99.4(4)	O6^{6}	Al1	O3	P1	-142.0(3)
O4	P2	O6	Al1^{3}	68.9(3)	O3^{5}	P1	O2	Al2^{1}	59.30(10)
O4	P2	O7	Al1^{4}	-173.5(3)	O3	P1	O2	Al2^{1}	-59.30(10)
O4	Al1	O3	P1	97.3(3)	O3^{5}	P1	O3	Al1	-70.5(4)
O5	P2	O4	Al1	-163.5(3)	O3	Al1	O4	P2	162.8(3)
O5	P2	O6	Al1^{3}	-170.0(3)	O7	P2	O4	Al1	76.8(4)
O5	P2	O7	Al1^{4}	66.8(3)	O7	P2	O5	Al2	20.7(4)
O5^{5}	Al2	O5	P2	72.7(4)	O7	P2	O6	Al1^{3}	-50.4(3)
O2	P1	O3	Al1	48.8(4)	O7^{1}	Al1	O4	P2	-77.2(4)
O2^{4}	Al2	O5	P2	-48.7(4)	O7^{1}	Al1	O3	P1	-21.2(4)
O6	P2	O4	Al1	-40.9(4)					

^{1}1+Y-X,2-X,+Z; ^{2}+Y-X,1-X,+Z; ^{3}1-Y+X,+X,1-Z; ^{4}2-Y,1+X-Y,+Z; ^{5}+X,+Y,3/2-Z; ^{6}+Y,1-X+Y,1-Z

Table 7 Solvent masks information for 17O2_sc2_0p43GPa.

Number	X	Y	Z	Volume	Electron count	Content
1	0.000	0.000	0.000	9.2	0.0	?
2	0.000	0.000	0.250	26.3	19.9	?
3	0.000	0.000	0.500	9.2	0.0	?
4	0.000	0.000	0.750	26.3	19.9	?
5	-0.610	-0.291	-0.525	904.6	228.6	?

Experimental

Single crystals of AlO_4P **[17O2_sc2_0p43GPa]** were **[]**. A suitable crystal was selected and **[]** on a **esperanto-CrysAlisPro-abstract goniometer imported esperanto images** diffractometer. The crystal was kept at 293(2) K during data collection. Using Olex2 [1], the structure was solved with the SHELXT [2] structure solution program using Intrinsic Phasing and refined with the SHELXL [3] refinement package using Least Squares minimisation.

1. Dolomanov, O.V., Bourhis, L.J., Gildea, R.J, Howard, J.A.K. & Puschmann, H. (2009), J. Appl. Cryst. 42, 339-341.
2. Sheldrick, G.M. (2015). Acta Cryst. A71, 3-8.
3. Sheldrick, G.M. (2015). Acta Cryst. C71, 3-8.

Crystal structure determination of [17O2_sc2_0p43GPa]

Crystal Data for AlO_4P (M =121.95 g/mol): hexagonal, space group P6$_3$/m (no. 176), a = 13.0719(5) Å, c = 15.3402(6) Å, V = 2270.1(2) Å^3, Z = 18, T = 293(2) K, μ(synchrotron) = 0.223 mm^{-1}, $Dcalc$ = 1.606 g/cm^3, 2825 reflections measured (3.118° ≤ 2Θ ≤ 31.956°), 1091 unique (R_{int} = 0.0200, R_{sigma} = 0.0249) which were used in all calculations. The final R_1 was 0.0332 (I > 2σ(I)) and wR_2 was 0.1041 (all data).

Refinement model description

Number of restraints - 0, number of constraints - unknown.

Details:

N/A

This report has been created with Olex2, compiled on 2023.03.06 svn.rbb2c1857 for OlexSys. Please let us know if there are any errors or if you would like to have additional features.
